# *Gaultheria leucocarpa* inhibits Aβ fibrillization and enhances mitophagy-mediated degradation of pathogenic proteins

**DOI:** 10.1016/j.neurot.2025.e00721

**Published:** 2025-08-14

**Authors:** Yue Zhang, Lan Deng, Jing Wei, Lufen Huang, Fei Gao, Lu Yu, Fengdan Zhu, Jianing Mi, Jianming Wu, Fang Ren, Minsong Guo, Xiaogang Zhou, Dalian Qin, Ting Chen, Anguo Wu

**Affiliations:** a Luzhou Key Laboratory of Activity Screening and Druggability Evaluation for Chinese Materia Medica, Key Laboratory of Medical Electrophysiology of Ministry of Education, Department of Cardiology, Department of Ophthalmology, The Affiliated Hospital of Southwest Medical University, School of Pharmacy, Southwest Medical University, Luzhou, 646000, China; b School of Pharmaceutical Sciences, China-Pakistan International Science and Technology Innovation Cooperation Base for Ethnic Medicine Development in Hunan Province, Hunan University of Medicine, Huaihua, 418000, China; c Department of Pharmacy, Jining Medical University, Rizhao 276500, China; d State Key Laboratory of Traditional Chinese Medicine Syndrome, The Second Affiliated Hospital of Guangzhou University of Chinese Medicine, Guangzhou, Guangdong, 510120, China; e Chongqing Key Laboratory of Sichuan-Chongqing Co-construction for Diagnosis and Treatment of Infectious Diseases Integrated Traditional Chinese and Western Medicine, Chongqing Traditional Chinese Medicine Hospital, Chongqing, 400021, China

**Keywords:** *Gaultheria leucocarpa*, Alzheimer's disease, Amyloid-beta, Mitophagy, *Caenorhabditis elegans*

## Abstract

Alzheimer's disease (AD) pathology involves amyloid-beta (Aβ) accumulation and neuronal toxicity, highlighting the need for therapeutic strategies that can both inhibit Aβ aggregation and promote pathogenic protein clearance. In this study, we identified *Gaultheria leucocarpa* as a medicinal plant with promising neuroprotective potential. Thioflavin T (ThT) fluorescence screening revealed that extracts from *G. leucocarpa* (GE), particularly the petroleum ether fraction of *G. leucocarpa* extract (GPF), effectively inhibited Aβ fibril formation *in vitro*. In cell-based assays, GPF significantly improved the viability of PC-12 ​cells exposed to Aβ peptides and fibrils, indicating protection against Aβ-induced cytotoxicity. Furthermore, GPF enhanced mitophagic activity, as demonstrated by increased GFP-LC3 puncta, elevated LC3-II/I ratio, and colocalization of GFP-LC3 with MitoTracker Red. Mechanistic investigations showed that GPF activates mitophagy via the AMPK/ULK1 pathway and inhibits the PI3K/AKT/mTOR pathway, resulting in enhanced degradation of APP and Tau proteins. In *Caenorhabditis elegans* models relevant to AD, GPF administration led to reduced Aβ deposits, delayed paralysis onset, improved food perception, and decreased oxidative stress. Collectively, these findings demonstrate that GPF exerts dual actions by inhibiting Aβ fibrillization and promoting mitophagy-mediated degradation of pathogenic proteins. The active ingredients identified from GPF extracts represent promising leads for the development of novel neuroprotective agents targeting AD-related pathological mechanisms.

## Highlights

• *Gaultheria leucocarpa* extract (GE), especially its GPF fraction, significantly inhibited amyloid-beta (Aβ) fibril formation.
• GPF improves the viability of PC-12 ​cells treated with Aβ peptides and fibrils.
• GPF activates mitophagy through the AMPK‒mTOR pathway, promoting the degradation of AD-related proteins.
• GPF alleviates AD-related pathology and behavioral deficits via mitophagy induction in *C. elegans.*

## Graphical abstract

**Figure:**
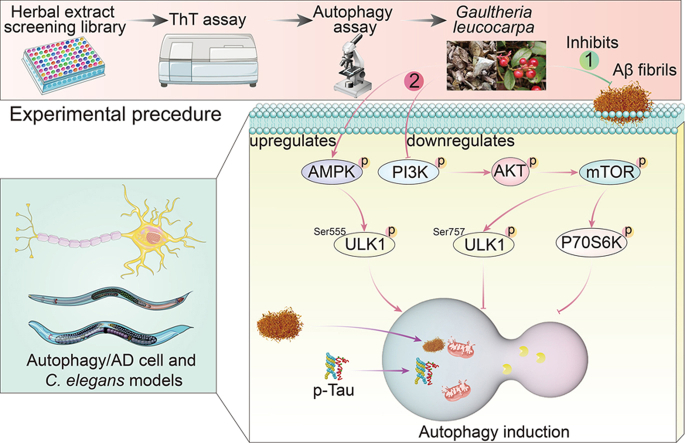

## Introduction

Alzheimer's disease (AD) is a progressive neurodegenerative disorder affecting millions globally. Recent estimates indicate that over 50 million people are currently living with AD, a number projected to triple by 2050 due to global population aging [1]. This rising prevalence highlights an urgent need for effective therapeutic strategies to address a growing public health crisis. Despite significant advances in our understanding of AD, it remains a formidable challenge in clinical practice. The disease is pathologically defined by extracellular amyloid-beta (Aβ) plaques and intracellular neurofibrillary tangles composed of hyperphosphorylated tau protein, both of which contribute to neurodegeneration and cognitive decline [[2], [3], [4], [5], [6]]. Traditional pharmacological interventions have largely targeted these hallmarks individually. However, the multifactorial nature of AD pathogenesis renders such monotargeted approaches insufficient [4,7,8]. The underlying pathophysiology of AD involves both aberrant protein aggregation and impaired protein clearance, suggesting that targeting a single mechanism often fails to yield meaningful clinical outcomes. Monotargeted therapies, such as those aimed solely at inhibiting Aβ aggregation or enhancing protein clearance, fall short in addressing the disease's complexity. As such, therapeutic strategies that restore proteostasis by both inhibiting protein aggregation and promoting protein degradation are increasingly regarded as essential.

Aβ peptides are generated via sequential cleavage of amyloid precursor protein (APP) by β-secretase and γ-secretase [[9], [10], [11]]. Among the resulting peptides, Aβ40 and Aβ42 are the most abundant, with Aβ42 being particularly prone to oligomerization and fibril formation, and thus more neurotoxic [12,13]. These peptides accumulate extracellularly to form plaques and initiate neuroinflammatory cascades via microglial and astrocytic activation, further exacerbating oxidative stress and neuronal damage [[14], [15], [16]]. Therapeutic strategies targeting Aβ include immunotherapies, aggregation inhibitors, and modulators of Aβ production [17].

Autophagy, a lysosome-mediated degradation pathway, is another promising target in AD therapy. This cellular process involves the formation of autophagosomes that sequester damaged proteins and organelles, which are subsequently degraded upon fusion with lysosomes [18,19]. Dysregulated autophagy has been increasingly implicated in AD, as it contributes to the accumulation of toxic Aβ and tau species [20,21]. Autophagy also facilitates the degradation of hyperphosphorylated tau, which disrupts microtubule stability and forms neurofibrillary tangles in AD [22]. Therefore, therapeutic strategies that both inhibit Aβ fibrillization and enhance autophagic clearance may offer a synergistic advantage in mitigating AD pathology [23].

Medicinal plants represent a rich resource for the development of multitarget therapeutics. Traditional Chinese Medicine (TCM), with its holistic, multicomponent, and multitarget treatment philosophy, is particularly well-suited for addressing complex diseases such as AD. In this study, we adopted a dual-targeting strategy to explore the potential of herbal extracts in both inhibiting Aβ fibrillization and promoting autophagic degradation of pathogenic proteins. Through comprehensive *in vitro* and in vivo screening, *G. leucocarpa* was identified as a promising candidate with dual activity. This plant, widely used in traditional medicine, has known anti-inflammatory, analgesic, antipyretic, and antimicrobial properties [[24], [25], [26], [27], [28]]. Its phytochemical profile includes flavonoids, phenolic acids, terpenoids, and essential oils [[29], [30], [31]]. However, its potential therapeutic effects against AD and underlying mechanisms remain unexplored.

## Materials and methods

### Reagents

3-(4,5-Dimethylthiazol-2-yl)-2,5-diphenyltetrazolium bromide (MTT, M2128), Hoechst 33342 (B2261), Thioflavin T (ThT, 596200), and hexafluoroisopropanol (HFIP, 105228) were purchased from Sigma‒Aldrich (St. Louis, MO, USA). Rapamycin (Rap, T1537), Compound C (CC, T1977), ULK-101 (T5403), and 3-methyladenine (3-MA, T1879) were obtained from TargetMol Co., Ltd. (MA, USA), and bafilomycin A1 (Baf, B101389) was sourced from Aladdin Bio-Chem Technology Co., Ltd. (Shanghai, China). Ultra-pure water was prepared using a Milli-Q Integral system (Millipore, Billerica, MA, USA). Methanol and acetonitrile were supplied by Adamas-beta (Shanghai, China). The Aβ1–42 peptide was purchased from China Peptides Co., Ltd. (Shanghai, China). Petroleum ether (L14649) and ethyl acetate (141-78-6) were also obtained from Sigma‒Aldrich. Antibodies against p-AKT (4060S), AKT (4691S), AMPK (2532S), p70S6K (2708S), p-AMPK (2535S), p-PI3K (4228L), PI3K (4257S), p-P70S6K (9234S), mTOR (2983S), p-ULK1 ​(Ser555) (14202S), and p-ULK1 (Ser555) (5869S) were obtained from Cell Signaling Technology, Inc. (CST, Beverly, MA, USA). The p-mTOR antibody (AP0115) was supplied by ABclonal Technology Co., Ltd. (Wuhan, China), and the ULK1 antibody (T56902) was from Abmart Shanghai Co., Ltd. (Shanghai, China). The 6E10 antibody (803001) was purchased from BioLegend (San Diego, CA, USA), and the GAPDH antibody (sc-47724)) was from Santa Cruz Biotechnology, Inc. (CA, USA). HRP-conjugated secondary antibodies, including anti-IgG (H ​+ ​L) mouse pAb-HRP (330) and rabbit pAb-HRP (458), were obtained from Medical & Biological Laboratories Co., Ltd. (MBL, Nagoya, Japan). The plasmids pEGFP-N1-APP (#69924), pRK5-EGFP-Tau-P301L (#46908), and pRK5-EGFP-Tau (#46904) were acquired from Addgene (Watertown, MA, USA). The Mito-QC plasmid was sourced from the Public Protein/Plasmid Library (PPL, Nanjing, China).

### Preparation of herbal extracts and fractions

The medicinal herbs used in this study were collected by our research team (Table S1) and authenticated by Professor Can Tang from Southwest Medical University. Voucher specimens (Nos. SW-202001 to SW-202060) have been deposited in the Department of Pharmacy at Southwest Medical University, Luzhou, China, for future reference. Methanol was selected as the extraction solvent due to its effectiveness in isolating a broad spectrum of bioactive compounds, including both polar and nonpolar constituents. For each herb, 500 ​g of dried and ground material was extracted twice with 4 ​L of methanol. The solvent was subsequently removed under reduced pressure to yield the crude methanol extract. Extraction yields for each herb are listed in Table S1. To generate fractions from the methanol extract of *G. leucocarpa* (GE), the crude extract was suspended in water and successively partitioned with petroleum ether, ethyl acetate, and n-butanol. This process resulted in four distinct fractions: petroleum ether fraction (GPF), ethyl acetate fraction (GEF), n-butanol fraction (GNF), and water fraction (GWF). Based on solvent polarity, the components of G. leucocarpa are distributed from least to most polar in the order of GPF, GEF, GNF, and GWF.

### Cell culture

Stable GFP-LC3 U87 ​cells, generously provided by Dr. Xiaoming Zhu from the Macau University of Science and Technology (Macao, China), were cultured in α-minimum essential medium (α-MEM) supplemented with 10 ​% fetal bovine serum (FBS, 164210, Procell), 50 U/mL penicillin, and 50 ​μg/mL streptomycin. PC-12 and SH-SY5Y cells, obtained from the American Type Culture Collection (ATCC, Virginia, USA), were maintained in complete Dulbecco's Modified Eagle Medium (DMEM, Gibco) supplemented with 10 ​% horse serum (HS, Gibco), 5 ​% FBS, 50 U/mL penicillin, and 50 ​μg/mL streptomycin. PC-12 and SH-SY5Y cells were selected due to their established role in neurodegenerative disease models, including AD, offering reproducibility and consistency across experimental conditions. All cell lines were cultured in a humidified incubator at 37 ​°C with 5 ​% CO_2_ and 75 ​% humidity.

### Preparation of Aβ1-42 fibril preparation and cell treatment

The preparation of Aβ1–42 fibrils followed a previously described protocol [32]. Briefly, 5 ​mg of Aβ1–42 peptide was dissolved in an appropriate volume of HFIP. The solution was aliquoted and evaporated under a stream of nitrogen to form thin peptide films, which were stored at −80 ​°C until use. For fibril formation, Aβ1–42 films were reconstituted in sterile phosphate-buffered saline (PBS) and incubated at 37 ​°C for 48 ​h. PC-12 cells were seeded in suitable culture plates and allowed to adhere. Cells were then treated with pre-formed Aβ1–42 fibrils at a final concentration of 20 ​μM for 24 ​h, either alone or in combination with test herbal extracts (200 ​μg/mL). Control groups received vehicle treatment only.

### MTT assay

Cell viability following treatment with herbal extracts or fractions was evaluated using the MTT assay. PC-12 or SH-SY5Y cells were seeded into 96-well plates at a density of 1 ​× ​10^4^ ​cells per well and allowed to adhere overnight. After 24 ​h, the medium was replaced with fresh DMEM containing various concentrations of the test compounds, followed by a 24 h incubation. Subsequently, 10 ​μL of MTT solution (5 ​mg/mL) was added to each well, and the plates were incubated for 4 ​h at 37 ​°C. After incubation, the MTT-containing medium was removed, and 100 ​μL of dimethyl sulfoxide (DMSO) was added to each well to dissolve the formazan crystals. Absorbance was measured at 570 ​nm using a Cytation 3 microplate reader (BioTek, VT, USA). Cell viability was calculated as a percentage of the absorbance relative to untreated control cells.

### Cell transfection

For transfection experiments, PC-12 or SH-SY5Y cells were seeded in 6-well plates at a density of 1 ​× ​10^5^ ​cells per well and allowed to adhere overnight. The plasmids used included pEGFP-N1-APP, pRK5-EGFP-Tau, pRK5-EGFP-Tau-P301L, and Mito-QC. Transfections were carried out using ExFect3000 Transfection Reagent (Vazyme, Nanjing, China) following the manufacturer's instructions. Briefly, 2 ​μg of plasmid DNA was diluted in 100 ​μL of serum-free DMEM, followed by the addition of 6 ​μL of ExFect3000 reagent. The mixture was gently vortexed and incubated at room temperature for 15 ​min to form DNA–transfection reagent complexes. These complexes were then added dropwise to each well containing PC-12 ​cells in 2 ​mL of serum-free DMEM. After 6 ​h of incubation, the medium was replaced with fresh DMEM supplemented with 10 ​% FBS. Cells were further incubated for 24 ​h to allow for transgene expression.

### Quantification of autophagy activity

Autophagy activity was quantified using stable GFP-LC3 U87 ​cells. Cells were seeded in 6-well plates at a density of 5 ​× ​10^4^ ​cells per well and allowed to adhere overnight. The following day, cells were treated with various concentrations of test compounds and incubated for 24 ​h. Rapamycin (Rap, 1 ​μμM) was used as a positive control for autophagy induction. After treatment, cells were washed twice with PBS and fixed with 4 ​% paraformaldehyde (PFA) for 15 ​min at room temperature. Following fixation, cells were rinsed with PBS and mounted using an anti-fade mounting medium. GFP-LC3 puncta, indicative of autophagosome formation, were visualized using a Zeiss Axio Observer fluorescence microscope (Carl Zeiss, Oberkochen, Germany). Autophagy activity was quantified by calculating the percentage of cells containing GFP-LC3 puncta. At least 100 ​cells were analyzed per condition in three independent experiments. Cells exhibiting five or more distinct GFP-LC3 puncta were classified as autophagy-positive. Data are presented as the percentage of autophagy-positive cells relative to the total number of cells counted.

### UHPLC-DAD-Q/TOF-MS/MS conditions

The chemical constituents of the GPF fraction were identified using ultra high-performance liquid chromatography coupled with diode-array detection and quadrupole time-of-flight tandem mass spectrometry (UHPLC-DAD-Q/TOF-MS/MS). Chromatographic separation was performed on a Shimadzu UHPLC system (Kyoto, Japan) using an Agilent Zorbax Eclipse Plus C18 column (1.8 ​μm, 100 ​mm ​× ​2.1 ​mm). The mobile phase consisted of solvent A (0.1 ​% formic acid in water) and solvent B (0.1 ​% formic acid in acetonitrile), delivered at a flow rate of 0.3 ​mL/min. The gradient elution program was as follows: 0–2 ​min, 10 ​% B; 2.01–12.5 ​min, 20 ​% B; 12.51–17.5 ​min, 40 ​% B; 17.51–25 ​min, 80 ​% B; 25.01–33 ​min, 95 ​% B; 33.01–35 ​min, 10 ​% B. The eluate was directed to a TripleTOF MS X500R system (AB SCIEX, Foster City, CA, USA) equipped with a DuoSpray ion source operating in negative electrospray ionization (ESI) mode. The ESI parameters were as follows: ion spray voltage, −4500 ​V; curtain gas, 35 psi; ion source temperature, 550 ​°C; declustering potential, −100 ​V; and both nebulizer and heater gases, 55 psi. Mass spectra were acquired over an *m*/*z* range of 100–1600 ​Da. Data acquisition and processing were performed using PeakView 1.4 software (AB SCIEX, Foster City, CA, USA).

### Thioflavin T assay

The Thioflavin T (ThT) assay was employed to monitor Aβ fibril formation, as it is a well-established method for detecting β-sheet-rich amyloid aggregates [33]. A freshly prepared 1 ​mM ​Aβ1–42 solution was mixed with PBS in the presence or absence of various test compounds, and the final volume of each reaction mixture was adjusted to 100 ​μL. At designated time points, 50 ​μL of ThT solution (20 ​μM, diluted in PBS, pH 7.4) was added to each 100 ​μL sample. The mixtures were transferred to black 96-well microplates suitable for fluorescence detection. Fluorescence intensities were measured using a BioTek fluorescence microplate reader (VT, USA) with an excitation wavelength of 450 ​nm and an emission wavelength of 485 ​nm. The fluorescence signal was directly proportional to the extent of Aβ fibril formation.

### Western blot

After treatment, cells were lysed in RIPA buffer (Cell Signaling Technology, USA) supplemented with protease and phosphatase inhibitor cocktails (TargetMol, Shanghai, China). Protein concentrations were determined using a BCA protein assay kit (Thermo Fisher Scientific, USA). Equal amounts of protein (20 ​μg per sample) were separated by 10–12 ​% SDS–PAGE and transferred onto polyvinylidene fluoride (PVDF) membranes (PALL, USA) using a wet transfer system (Bio-Rad, Hercules, CA, USA). Membranes were blocked with 5 ​% nonfat milk in Tris-buffered saline containing 0.1 ​% Tween-20 (TBST) for 1 ​h at room temperature. Following blocking, membranes were incubated overnight at 4 ​°C with the appropriate primary antibodies. After washing three times with TBST, membranes were incubated with HRP-conjugated secondary antibodies (1:5000; Cell Signaling Technology, USA) for 1 ​h at room temperature. After a final series of washes, immunoreactive bands were visualized using an Supersensitive chemiluminescence (ECL) detection reagent (Oriscience, China) and imaged using a ChemiDoc MP Imaging System (Bio-Rad, USA). Band intensities were quantified using ImageJ software (National Institutes of Health, USA).

### Molecular docking

The molecular structures of the selected ligands, including Dhasingreoside, Gaultherin, and (7R,8S,8′S)-10-O-Benzoyl-isolariciresinol, were initially retrieved in SMILES format and converted into 3D SDF files using the Novopro online tool (https://www.novopro.cn/tools/mol2smiles.html). The three-dimensional structures of the target proteins AMPK1 (PDB ID: 6C9H), PIK3R1 (PDB ID: 4JPS), and APP (PDB ID: 1AAP) were obtained from the RCSB Protein Data Bank (https://www.rcsb.org/). The structure of the PINK1 protein (UniProt ID: Q9BXM7) was predicted using AlphaFold (AF-Q9BXM7-F1) and downloaded from the AlphaFold Protein Structure Database (https://alphafold.ebi.ac.uk/). Molecular docking simulations were conducted using AutoDock Tools version 1.5.6 to evaluate the binding affinities between the ligands and target proteins. The resulting ligand–protein complexes were further analyzed and visualized using PyMOL software.

### C. elegans strains and maintenance culture

The *C. elegans* strains used in this study included DA2123 [*lgg-1*p::GFP:*lgg-1* ​+ ​rol-6 (su1006)], BC12921 [rCes T12G3.1:GFP ​+ ​pCeh361], IR1631: N2; Ex003 [pmyo-3TOMM-20:Rosella; pRF4], CL2006 ((dvIs2 [pCL12 (unc-54/human Aβ1–42 minigene) ​+ ​pRF4]), CL4176 (dvIs27 [myo-3p:A-Beta (1–42):let-851 30UTR) ​+ ​rol-6(su1006)], CL2122 (dvIs15 [(pPD30.38) unc-54 (vector) ​+ ​(pCL26) mtl-2:GFP], and CL2355 (dvIs50 [pCL45 (snb-1:A-beta 1–42:3′ UTR(long) ​+ ​mtl-2:GFP]. All strains were obtained from the Caenorhabditis Genetics Center (CGC, University of Minnesota, Minneapolis, MN, USA). Worms were maintained on nematode growth medium (NGM) agar plates seeded with *Escherichia coli* OP50 and cultured at 20 ​°C under standard laboratory conditions.

### Determination of autophagy in C. elegans

The *C. elegans* strains DA2123 and BC12921 were used to evaluate autophagy [34]. DA2123 expresses GFP-tagged LGG-1, a homolog of mammalian LC3, in body wall muscle and intestinal cells. BC12921 expresses a GFP-tagged p62/SQST-1 fusion protein. Synchronized L4 larvae were transferred to NGM plates supplemented with either the GPF fraction or Rap as a positive control. Control plates contained 0.1 ​% DMSO. After treatment, worms were washed with M9 buffer and immobilized on 2 ​% agarose pads containing 1 ​mM sodium azide. Autophagy was assessed by examining GFP::LGG-1 puncta in the body wall muscle cells of DA2123 and by analyzing p62:GFP expression in BC12921 using a Leica DM6B fluorescence microscope (Leica, Germany). For DA2123, autophagosomes were quantified by counting the number of GFP::LGG-1 puncta in at least 20 randomly selected cells per worm, with a minimum of 20 worms per group. The mean number of puncta per cell was used as an index of autophagy activity. For BC12921, GFP intensity indicating p62 expression was quantified using ImageJ software. All experiments were conducted in triplicate.

### Determination of mitophagy in C. elegans

The *C. elegans* strain IR1631, which expresses the mitophagy reporter TOMM-20:Rosella in body wall muscle cells, was kindly provided by Dr. Tavernarakis and used for mitophagy assessment. To synchronize the population, gravid adults were treated with a bleaching solution containing 0.5 ​M NaOH and 1 ​% NaOCl to isolate eggs. The eggs were then incubated overnight in M9 buffer to allow hatching and obtain synchronized L1 larvae. L1 larvae were transferred to fresh NGM plates and cultured until the L4 stage. Synchronized L4 larvae were then transferred to NGM plates containing either the GPF fraction or UA. After treatment, worms were washed off the plates with M9 buffer and immobilized on 2 ​% agarose pads containing 1 ​mM sodium azide. Mitophagy was evaluated by visualizing TOMM-20:Rosella puncta formation in the mitochondria of body wall muscle cells using fluorescence microscopy. The Rosella probe is a pH-sensitive dual-emission biosensor that enables the detection of mitophagy by differentially emitting fluorescence under neutral and acidic conditions. Quantitative analysis was performed by calculating the fluorescence intensity ratio of pH-sensitive GFP to pH-insensitive DsRed using ImageJ software, according to previously described protocols [33,35].

### Paralysis analysis

The *C. elegans* strains used for paralysis analysis were CL2006 and CL4176 [36], both of which express human Aβ1–42 in body wall muscle cells and display paralysis as a measurable phenotype. For synchronization, gravid adults were treated with a bleaching solution (0.5 ​M NaOH and 1 ​% NaOCl) to isolate eggs, which were then incubated overnight in M9 buffer to obtain synchronized L1 larvae. These larvae were transferred to fresh NGM plates and cultured to the L4 stage. Synchronized L4 larvae were then placed on NGM plates containing various concentrations of the GPF fraction. Control plates contained an equivalent volume of DMSO (0.1 ​%). For the temperature-sensitive CL4176 strain, paralysis was induced by shifting the incubation temperature from 16 ​°C to 25 ​°C for 24 ​h. In contrast, the constitutively expressing CL2006 strain was maintained at 20 ​°C throughout the experiment. Paralysis was assessed by gently prodding worms with a platinum wire at regular intervals. Worms were scored as paralyzed if they failed to respond to touch or exhibited only head movement. The number of paralyzed worms was recorded starting at 36 ​h post-treatment and monitored until the entire population became immobile. Each experimental condition was performed in triplicate, with a minimum of 150 worms per replicate to ensure statistical validity.

### Aβ3-42 aggregation analysis

Aggregation of the Aβ3-42 peptide in *C. elegans* was assessed using the transgenic strain CL2331, which expresses a GFP-tagged human Aβ3-42 fusion protein in body wall muscle cells [36,37]. In this temperature-sensitive model, Aβ3-42 aggregation is endogenously induced by shifting worms to 25 ​°C, which activates transgene expression and results in the formation of visible green fluorescent aggregates within muscle tissue. Synchronized CL2331 worms were treated with the GPF fraction and maintained at 25 ​°C until the second day of adulthood. No exogenous Aβ peptides were introduced during the experiment. After treatment, worms were immobilized on glass slides using 0.1 ​% sodium azide (NaN_3_) and imaged under a fluorescence microscope (Leica, Germany). Quantification of Aβ3-42 aggregates was performed by counting fluorescent puncta in the anterior region of each worm using ImageJ software.

### Food-sensing behavior assay

The *C. elegans* strains used for the food-sensing behavior assay included CL2355 and its vector control CL2122, which express human Aβ in neurons, as well as BR5270 and its vector control BR5271, which express the proaggregating F3ΔK280 human Tau protein panneuronally [38]. Worms were cultured on E. coli OP50-seeded plates (8 ​cm diameter with a 1 ​cm inner unseeded area). After 48 ​h of GPF treatment, food-sensing behavior was assessed as previously describe [38]. Briefly, treated and control worms were washed three times with M9 buffer to remove residual bacteria and transferred to unseeded NGM plates for 1 ​h to standardize gut content prior to subsequent analysis. Following the starvation period, individual worms were placed in the center of an assay plate—an 8 ​cm NGM plate with a ring of E. coli OP50 located 1 ​cm from the edge. After a 5-min acclimation period, the number of body bends was recorded over a 20-s interval. Food-sensing behavior was quantified based on the slowing rate of body bending in response to bacterial presence. The slowing rate was calculated using the formula: Slowing rate = (N without food – N with food)/N without food, where N is the number of body bends in the absence or presence of food. Each experimental condition included a minimum of 20 worms and was repeated in triplicate to ensure statistical reliability.

### DHE staining

Reactive oxygen species (ROS) levels in *C. elegans* were quantified using a dihydroethidium (DHE) staining protocol, as previously described [34]. Strains CL4176, BR5270, and BR5271 were treated with the GPF fraction and subsequently collected. Worms were washed three times with M9 buffer to remove residual bacteria and debris. After cleaning, worms were incubated in 100 ​μM DHE at 20 ​°C for 1 ​h in the dark to allow for selective staining of ROS. Following incubation, worms were mounted on 2 ​% agarose pads and immobilized with 10 ​mM sodium azide. Fluorescence imaging was performed using a fluorescence microscope, and red fluorescence protein (RFP, indicative of oxidized DHE) was measured to assess ROS levels. Quantification of fluorescence intensity was carried out using ImageJ software, providing a measure of ROS accumulation in treated versus control groups.

### RNA interference

RNA interference (RNAi) was employed to examine gene-specific effects in *C. elegans* by feeding worms bacteria engineered to express double-stranded RNA targeting specific genes [39]. Bacterial strains producing RNAi constructs against *lgg-1*, *vps-34*, and *unc-5*1, along with the control strain HT115 carrying the empty L4440 vector, were cultured overnight in LB medium containing ampicillin and tetracycline. The cultured bacteria were then seeded onto NGM plates supplemented with 1 ​mM isopropyl β-*d*-1-thiogalactopyranoside (IPTG), 100 ​μg/mL ampicillin, and 12.5 ​μg/mL tetracycline to induce dsRNA expression. After induction, synchronized L1-stage CL4176 worms were transferred onto the RNAi plates to initiate gene knockdown through feeding. All RNAi bacterial strains were sourced from the Ahringer RNAi library, ensuring standardized and validated gene silencing protocols.

### Statistical analysis

Statistical analyses were performed using GraphPad Prism version 8.0 (GraphPad Software, USA). Data are presented as mean ​± ​standard error of mean (SEM) from a minimum of three independent experiments. One-way analysis of variance (ANOVA) followed by Tukey's post hoc test was used to determine statistical significance. A p-value of less than 0.05 was considered statistically significant.

## Results

### *In vitro* screening of herbal extracts targeting Aβ inhibition

**Fig. 1:**
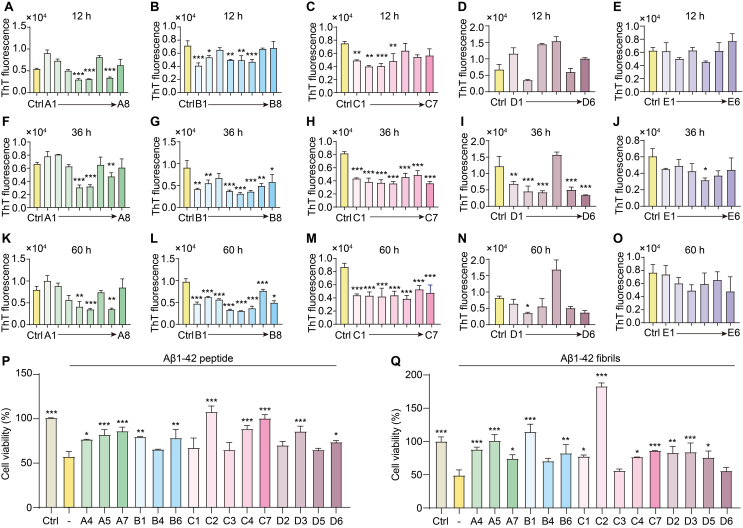
*In vitro* screening of herbal extracts for inhibition of amyloid-beta (Aβ) fibril formation. Thioflavin T (ThT) fluorescence assays were performed to assess the inhibitory effects of various herbal extracts (200 μg/mL) on a fibril formation at three incubation times: 12 h, 36 h, and 60 h. Treatment groups included extracts labeled A1–A8 (green), B1–B8 (blue), C1–C7 (pink), D1–D6 (brown), and E1–E6 (purple). (**A-E**) ThT fluorescence after 12 h of incubation. (**F-J**) ThT fluorescence after 36 h of incubation. (**K–O**) ThT fluorescence after 60 h of incubation. (**P**) Cell viability of PC-12 cells treated with Aβ1‒42 peptide alone (−) or co-treated with herbal extracts (200 μg/mL). (**Q**) Cell viability of PC-12 cells treated with pre-formed Aβ1‒42 fibrils alone (−) or co-treated with herbal extracts (200 μg/mL). Data are presented as the means ± SEM. ∗*p* < 0.05, ∗∗*p* < 0.01, ∗∗∗*p* < 0.001 vs. the Ctrl group.

To identify herbal extracts capable of inhibiting Aβ fibrillization, we performed ThT fluorescence assays at 12, 36, and 60 ​h of incubation. ThT fluorescence intensity, which correlates with Aβ fibril formation, was measured at each time point. All extracts were tested at 200 ​μg/mL. Several extracts showed significant inhibitory effects on fibrillization. At 12 ​h, extracts A4, A5, A7, B1, B2, B4-B6, and C1-C4 markedly reduced ThT fluorescence (Fig. 1A–E). By 36 ​h, extracts A4, A5, A7, B1, B2, B4–B8, C1–C7, D1–D3, D5, D6, and E4 continued to demonstrate inhibition (Fig. 1F–J). After 60 ​h, A4, A5, A7, B1–B8, C1–C7, and D2 maintained significant effects (Fig. 1K-O). To assess the cytoprotective potential, PC-12 ​cells were treated with Aβ1–42 peptide and fibrils, in the presence of the same extracts at their maximum non-toxic concentrations (200 ​μg/mL for A4–A7, B1, C1–C7, D3; 50 ​μg/mL for B4–B6, D2, D5–D6). Several extracts significantly improved cell viability compared to Aβ1–42 alone. Notably, A4, A5, A7, B1, B6, C2, C4, C7, D2, D3, D5, and D6 showed substantial protective effects (Fig. S1 and Fig. 1P–Q). These findings highlight specific herbal extracts that effectively inhibit Aβ fibrillization and increase cell viability, suggesting their potential as neuroprotective agents against Aβ-induced toxicity.

### Screening potential autophagy activators in vitro

**Fig. 2:**
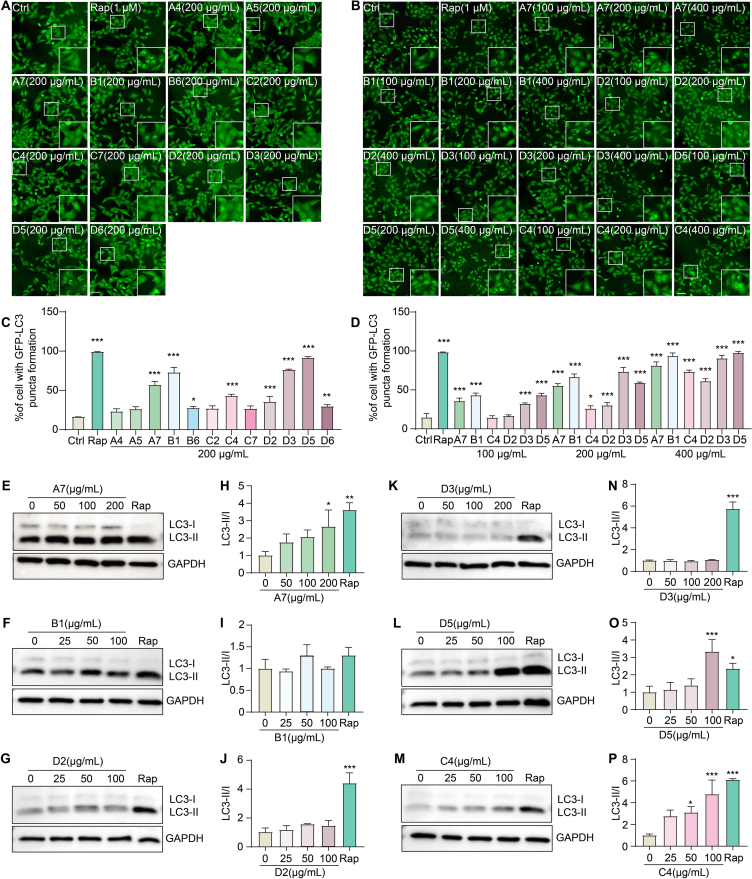
Screening potential autophagy activators *in vitro*. (**A**) Representative fluorescence microscopy images of stable GFP-LC3 U87 cells treated with herbal extracts (200 μg/mL), including A4, A5, A7, B1, B6, C2, C4, C7, D2, D3, D5, and D6. Cells treated with rapamycin (Rap, 1 μM) served as a positive control. White boxes indicate enlarged regions showing increased GFP-LC3 puncta. Magnification: × 20; scale bar: 100 μm. (**B**) Representative fluorescence microscopy images of stable GFP-LC3 U87 cells after treatment with herbal extracts (100, 200, and 400 μg/mL), including A7, B1, C4, D2, D3, and D5. Cells treated with Rap (1 μM) served as a positive control. White boxes indicate enlarged regions showing increased GFP-LC3 puncta. Magnification: × 20; scale bar: 100 μm. (**C**) Quantification of GFP-LC3 puncta formation in stable GFP-LC3 U87 cells treated with Rap, A4, A5, A7, B1, B6, C2, C4, C7, D2, D3, D5, and D6. Data are presented as means ± SEM. ∗*p* < 0.05, ∗∗*p* < 0.01, ∗∗∗*p* < 0.001 vs. the Ctrl group. (**D**) Quantification of GFP-LC3 puncta formation in stable GFP-LC3 U87 cells treated with Rap, A7, B1, C4, D2, D3, and D5. Data are presented as means ± SEM. ∗*p* < 0.05, ∗∗∗*p* < 0.001 vs. the Ctrl group. (**E**-**G**, **K**-**M**) Western blot analysis of LC3-II/I conversion in PC-12 cells treated with the herbal extracts A7, B1, D2, D3, D5, and C4 at various concentrations. GAPDH was used as a loading control. Original unprocessed Western blots are shown in Fig. S8. (**H**-**J**, **N**–**P**) Quantification of the LC3-II/I ratios from Western blot analyses. Data are presented as means ± SEM. ∗*p* < 0.05, ∗∗*p* < 0.01, ∗∗∗*p* < 0.001 vs. the Ctrl group.

Activation of autophagy has emerged as a promising therapeutic strategy for AD [40]. To determine which Aβ-inhibitory herbal extracts also promote autophagy, we evaluated autophagic responses via GFP-LC3 puncta formation in stable GFP-LC3 U87 ​cells and LC3-II/I conversion in PC-12 ​cells. The autophagy-inducing potential of each extract was assessed relative to Rap, a standard positive control. Fluorescence microscopy revealed that extracts A7, B1, B6, C4, D2, D3, D5, and D6 significantly increased GFP-LC3 puncta at 200 ​μg/mL (Fig. 2A–C). Based on these results, the most active extracts (A7, B1, C4, D2, D3, and D5) were further evaluated at multiple concentrations (100, 200, and 400 ​μg/mL). These extracts demonstrated a dose-dependent increase in GFP-LC3 puncta formation (Fig. 2B–D). Western blot analysis confirmed that A7, D5, and C4 notably elevated the LC3-II/I ratio in a dose-dependent manner, indicating enhanced autophagic flux (Fig. 2E-P). Among these, extract A7 (*G. leucocarpa*) exhibited both strong Aβ1-42 inhibitory activity and robust autophagy induction, and was therefore selected for further investigation.

### GPF inhibits Aβ fibrillization and cytotoxicity *in vitro*

**Fig. 3:**
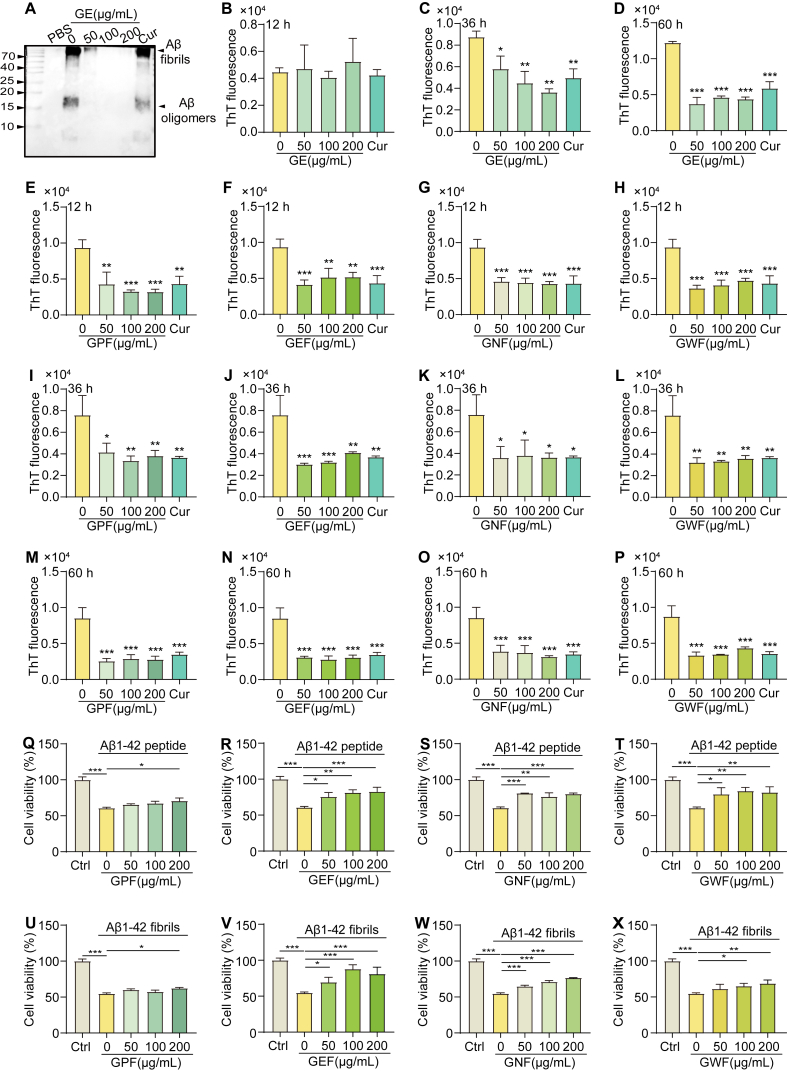
*G. leucocarpa* petroleum ether fraction (GPF) inhibits Aβ fibrillization and cytotoxicity *in vitro*. (**A**) Western blot analysis showing the effect of extract of *G. leucocarpa* (GE) on Aβ fibrils and oligomers in PC-12 cells treated with GE at concentrations of 50, 100, or 200 μg/mL. Curcumin (Cur, 20 μM) was used as a positive control. (**B**–**D**) ThT fluorescence assay assessing Aβ fibrillization after 12, 36, and 60 h of incubation with various concentrations of G. (**E**–**H**) ThT fluorescence assay after 12 h of incubation with different fractions of *G. leucocarpa*: GPF, GEF, GNF, and GWF. (**I**–**L**) ThT fluorescence assay after 36 h of incubation with the same fractions. (**M**–**P**) ThT fluorescence assay after 60 h of incubation with the same fractions. (**Q**–**T**) Viability of PC-12 cells treated with Aβ1‒42 peptide and various concentrations of GPF, GEF, GNF, or GWF. (**U**–**X**) Viability of PC-12 cells treated with Aβ1‒42 fibrils and the same concentrations of each fraction. Data are presented as means ± SEM. ∗*p* < 0.05, ∗∗*p* < 0.01, ∗∗∗*p* < 0.001 vs. the Ctrl group.

The anti-amyloidogenic activity of GE was further evaluated *in vitro* by Western blotting analysis using the 6E10 antibody. As shown in Fig. 3A, GE treatment at concentrations of 50, 100, and 200 ​μg/mL, as well as curcumin (Cur, 20 ​μM) as a positive control, significantly reduced levels of Aβ fibrils and oligomers, suggesting GE's potential to inhibit Aβ aggregation. ThT fluorescence assays were conducted to evaluate the fibrillization kinetics of Aβ in the presence of GE and its fractions (GPF, GEF, GNF, and GWF) over 12, 36, and 60 ​h of incubation. GE reduced ThT fluorescence in a dose-dependent manner across all time points (Fig. 3B–D), indicating inhibition of fibril formation. All four fractions similarly demonstrated significant inhibition of ThT fluorescence at multiple concentrations and incubation times: GPF, GEF, GNF, and GWF consistently suppressed fibrillization at 12 ​h (Fig. 3E–H), 36 ​h (Fig. 3I-L), and 60 ​h (Fig. 3M−P). Western blot analysis further validated the inhibitory effects of GPF on Aβ fibrils and oligomers (Fig. S2). To evaluate cytoprotective effects, PC-12 ​cells were exposed to Aβ1-42 peptide and fibrils in the presence of the GE fractions. Treatment with GPF, GEF, GNF, or GWF significantly improved cell viability in a dose-dependent manner (Fig. 3Q-T). Consistent protective effects were also observed in the presence of preformed Aβ1–42 fibrils (Fig. 3U–X). Additionally, experiments in human neuronal SH-SY5Y cells corroborated these results. GPF treatment markedly increased cell viability and reduced the percentage of PI-positive cells following exposure to Aβ1–42 fibrils (Fig. S5), reinforcing the protective capacity observed in PC-12 ​cells. In summary, GPF and other fractions of *G. leucocarpa* effectively inhibited Aβ fibrillization and attenuated Aβ-induced cytotoxicity, highlighting their potential as neuroprotective agents.

### GPF activates mitophagy *in vitro*

**Fig. 4:**
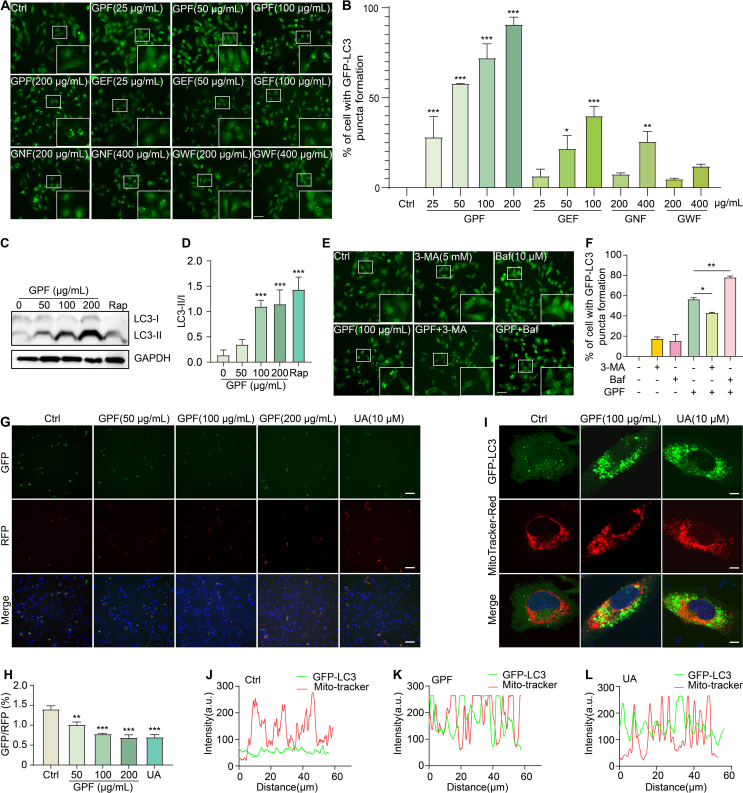
GPF activates mitophagy *in vitro*. (**A**) Representative fluorescence microscopy images of GFP-LC3 puncta in stable GFP-LC3 U87 cells treated with various concentrations of GPF, GEF, GNF, or GWF. White boxes highlight regions with increased puncta. Magnification: × 20; scale bar: 100 μm. (**B**) Quantification of GFP-LC3 puncta formation in cells treated with different concentrations of GPF, GEF, GNF, or GWF. Data are presented as means ± SEM. ∗*p* < 0.05, ∗∗*p* < 0.01, ∗∗∗*p* < 0.001 vs. the Ctrl group. (**C**, **D**) Western blot analysis of LC3-II/I conversion in PC-12 cells treated with various concentrations of GPF. GAPDH served as a loading control. Data are presented as means ± SEM. ∗∗∗*p* < 0.001 vs. the Ctrl group. Original unprocessed Western blots are shown in Fig. S9. (**E**, **F**) Fluorescence microscopy and quantification of GFP-LC3 puncta in stable GFP-LC3 U87 cells treated with GPF (100 μg/mL) alone or in combination with 3-MA (5 mM) or Baf (10 μM). White boxes indicate enlarged regions showing increased GFP-LC3 puncta. Magnification: × 20; scale bar: 100 μm. Data are presented as means ± SEM. ∗*p* < 0.05, ∗∗*p* < 0.01. (**G**) Representative images showing GFP and RFP signals in PC-12 cells transfected with the Mito-QC plasmid and treated with different concentrations of GPF or UA (10 μM). Magnification: × 20; scale bar: 100 μm. (**H**) Quantification of the GFP/RFP fluorescence ratio in Mito-QC–expressing PC-12 cells treated with GPF or UA. Data are presented as means ± SEM. ∗∗*p* < 0.01, ∗∗∗*p* < 0.001 vs. the Ctrl group. (**I**) Fluorescence microscopy images showing colocalization of GFP-LC3 and MitoTracker Red in PC-12 cells treated with GPF (100 μg/mL) or UA (10 μM). Magnification: × 63; scale bar: 100 μm. (**J**–**L**) Line scan analysis of GFP-LC3 and MitoTracker Red fluorescence intensity in PC-12 cells treated with Ctrl, GPF, or UA. Overlapping fluorescence profiles indicate mitophagy induction.

To investigate the potential of *G. leucocarpa* fractions in mitophagy activation, we performed a series of assays using stable GFP-LC3 U87 ​cells and PC-12 ​cells. Among all tested fractions, GPF exhibited the strongest induction of GFP-LC3 puncta formation in a dose-dependent manner (Fig. 4A and B). Western blot analysis confirmed that GPF treatment significantly increased the LC3-II/I ratio in PC-12 ​cells, an effect comparable to that observed with Rap (Fig. 4C and D). To assess autophagic flux, autophagy inhibitors 3-MA and Baf were used. As expected, 3-MA reduced GFP-LC3 puncta, while Baf increased puncta formation, validating the dynamic regulation of autophagy by GPF (Fig. 4E and F). Mitophagy was further evaluated in PC-12 ​cells transfected with the Mito-QC construct. GPF treatment led to a dose-dependent decrease in the GFP/RFP ratio, consistent with enhanced mitophagy, and showed effects comparable to those of the positive control UA (Fig. 4G and H). Colocalization analysis of GFP-LC3 and MitoTracker Red revealed significant overlap in fluorescence signals following GPF treatment, as shown by intensity profile line scans, further supporting mitophagy induction (Fig. 4I-L). Chemical profiling of GPF using UHPLC-DAD-QTOF-MS/MS identified several potential bioactive constituents, including miquelianin, gaultherin, and gaultherin A (Fig. S3 and Table 1). Collectively, these findings demonstrate that GPF robustly activates mitophagy *in vitro*, potentially through multiple bioactive compounds.

**Table 1:** UHPLC-DAD-QTOF-MS/MS analysis of GPF in negative ion mode.

NO.	RT (min)	Chemical name	Chemical formula	Molecular weight	[M − H]^-^
1	20.4	Gaultherin	C_19_H_26_O_12_	446	445.1
2	20.4	9-*O*-Benzoyl-lariciresinol	C_27_H2_8_O_7_	464	463.1
3	21.4	Gaultheric acid	C_19_H_24_O_4_	316	315.2
4	21.6	Pernetic acid D	C_15_H_22_O_4_	266	265.1
5	21.8	4′,5-Dihydroxy-7-methoxy-6-methylflavone,8-Demethylsideroxylin	C_17_H_14_O_5_	298	297.1
6	22.5	Dhasingreoside	C_27_H_28_O_18_	640	639.1
7	23.6	5-Hydroxy-4′,7-dimethoxy-6-methylflavone	C_18_H_16_O_5_	312	311.1
8	23.8	4′,5-Dihydroxy-3,7-dimethoxy-6-methylflavone(8-Demethyllatifolin)	C_18_H_16_O_6_	328	327.1
9	24.8	Gaultheroside A	C_27_H_36_O_12_	552	551.2
10	24.9	Arbutin	C_12_H_16_O_7_	272	271.1
11	25.0	Gaultherin A	C_25_H_30_O_9_	474	473.2
12	26.7	Gaultherin B	C_26_H_32_O_10_	504	503.2
13	34.2	(7R,8S,8′S)-9-O-Benzoyl-isolariciresinol	C_27_H_28_O_7_	464	463.2

### Analysis of potential molecular targets

**Fig. 5:**
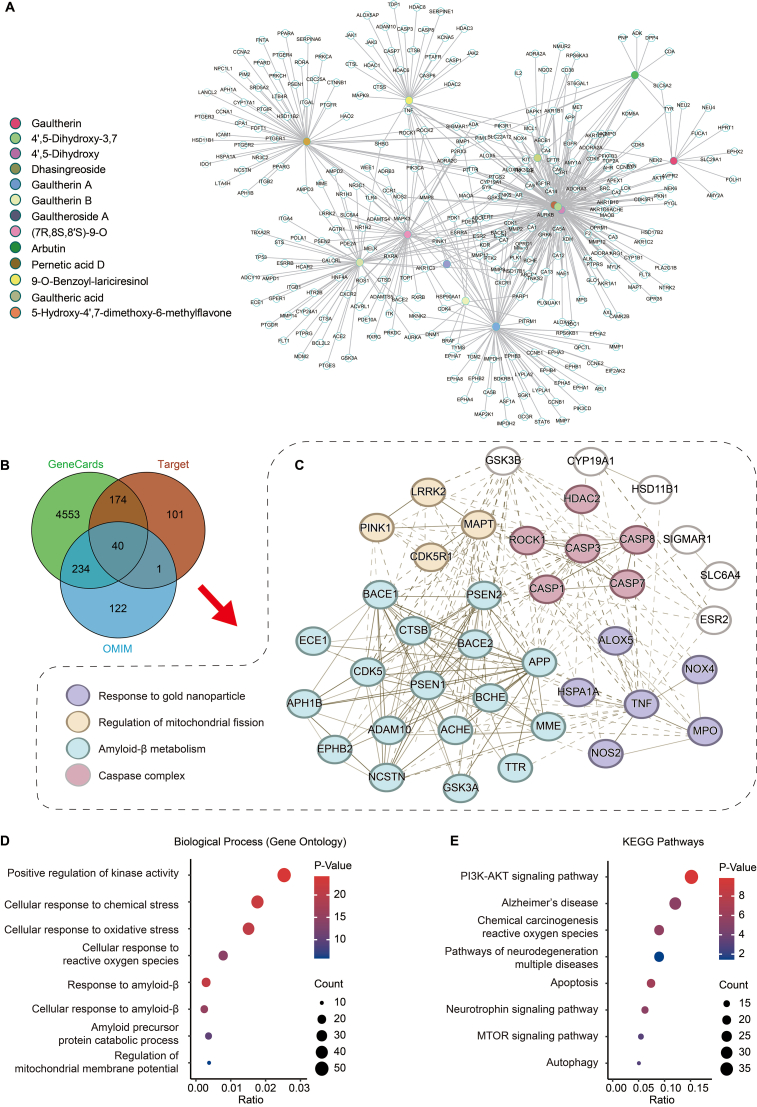
Analysis of potential molecular targets. (**A**) Compound–target interaction network for 13 identified compounds in GPF, illustrating the predicted associations with putative protein targets. (**B**) Venn diagram showing the overlap among 316 predicted targets of the 13 GPF compounds, 5001 Alzheimer's disease (AD)-related genes from the GeneCards database, and 397 AD-related genes from the OMIM databas. (**C**) Protein–protein interaction (PPI) network of the 40 overlapping genes between GPF compound targets and AD-related genes, with pathways relevant to AD highlighted. (**D**) Gene Ontology (GO) biological process (BP) enrichment analysis of the 40 shared genes, revealing key biological processes potentially influenced by GPF. (**E**) KEGG pathway enrichment analysis of the same shared gene set, identifying signaling pathways potentially involved in the anti-AD effects of GPF.

Mass spectrometry analysis identified 13 distinct compounds within the GPF fraction. To elucidate their molecular mechanisms, we employed SwissTargetPrediction to predict potential targets, yielding 316 unique protein targets (Fig. 5A and Table S2). Concurrently, AD-related genes were compiled from the GeneCards (n ​= ​5001) and OMIM (n ​= ​397) databases. Cross-referencing these datasets revealed 40 overlapping genes between the predicted compound targets and AD-associated genes (Fig. 5B). Protein–protein interaction (PPI) network analysis of these 40 overlapping targets demonstrated a densely interconnected network. MCODE clustering further indicated that these targets are significantly enriched in pathways associated with Aβ formation, apoptotic regulation, and mitochondrial fission (Fig. 5C). These findings are consistent with our experimental data showing GPF's inhibition of Aβ aggregation and activation of mitophagy.

Gene Ontology (GO) enrichment analysis of the overlapping genes highlighted key biological processes, including positive regulation of kinase activity, responses to oxidative and chemical stress, cellular responses to Aβ, APP catabolic processes, and regulation of mitochondrial membrane potential (Fig. 5D). KEGG pathway analysis revealed that the most enriched pathways included the PI3K-AKT signaling pathway, AD pathology, ROS-related carcinogenesis, neurodegenerative disease pathways, apoptosis, neurotrophin signaling, mTOR signaling, and autophagy (Fig. 5E).

To further validate these predictions, molecular docking simulations were performed using key bioactive compounds from GPF and their top-ranked target proteins (Table S3). Among these, three major active compounds, including (7R,8S,8′S)-9-O-benzoyl-isolariciresinol, dhasingreoside, and gaultherin, were selected for detailed docking studies to assess their potential biological activities. Notably, (7R,8S,8′S)-9-O-benzoyl-isolariciresinol and dhasingreoside exhibited strong binding affinities toward AMPK, PI3K, and PINK1, which are pivotal regulators of autophagy and mitophagy. In contrast, gaultherin showed a high binding affinity for APP, suggesting its potential role in inhibiting Aβ aggregation (Fig. S4). Collectively, these computational analyses illuminate the compound–target landscape of GPF and support its multifaceted therapeutic potential in AD by modulating autophagy, mitophagy, and Aβ pathology.

### GPF activates mitophagy via the AMPK‒mTOR pathway

**Fig. 6:**
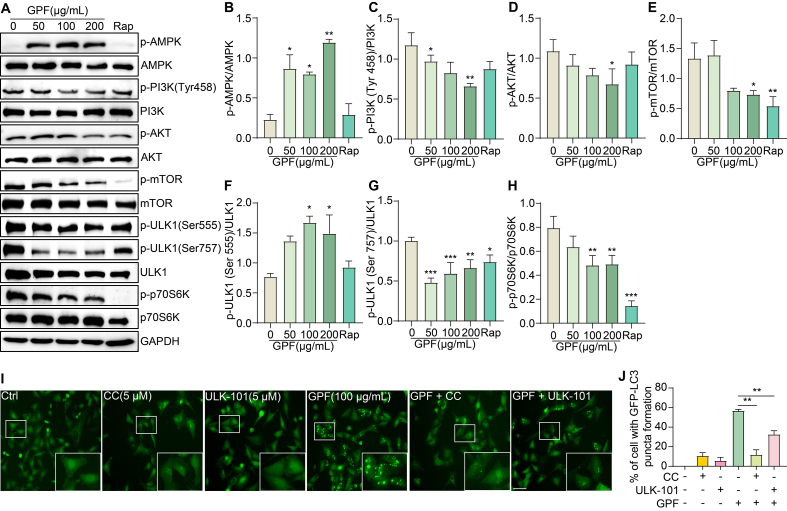
GPF activates mitophagy via the AMPK‒mTOR pathway. (**A**) Western blot analysis of key proteins in the AMPK/ULK1 and PI3K/AKT/mTOR signaling pathways in PC-12 cells treated with GPF at concentrations of 50, 100, or 200 μg/mL. Rap (1 μM) was used as a positive control. Analyzed proteins include *p*-AMPK, AMPK, p-PI3K (Tyr458), PI3K, *p*-AKT, AKT, *p*-mTOR, mTOR, *p*-ULK1 (Ser555), *p*-ULK1 (Ser757), ULK1, p-p70S6K, and p70S6K. GAPDH served as a loading control. Original unprocessed blots are shown in Fig. S10. (**B**–**H**) Quantification of the phosphorylated-to-total protein ratios for AMPK, PI3K, AKT, mTOR, ULK1 (Ser555), ULK1 (Ser757), and p70S6K. Data are presented as mean ± SEM. ∗*p* < 0.05, ∗∗*p* < 0.01, ∗∗∗*p* < 0.001 vs. the Ctrl group. (**I**) Representative fluorescence microscopy images showing GFP-LC3 puncta in PC-12 cells treated with GPF (100 μg/mL) alone or in combination with compound C (CC, 5 μM) or ULK-101 (5 μM). White boxes indicate enlarged regions showing increased GFP-LC3 puncta. Magnification: × 20; scale bar: 100 μm. (**J**) Quantification of GFP-LC3 puncta in PC-12 cells treated with GPF alone or combined with CC or ULK-101. Data are shown as mean ± SEM. ∗∗*p* < 0.01.

To validate the predicted molecular targets and elucidate the signaling mechanism underlying GPF-induced mitophagy, we examined the involvement of the AMPK/ULK1 and PI3K/AKT/mTOR pathways in PC-12 ​cells. Cells were treated with varying concentrations of GPF (50, 100, and 200 ​μg/mL), with Rap (1 ​μM) serving as a positive control. Western blot analyses demonstrated that GPF treatment resulted in a dose-dependent increase in the phosphorylation of AMPK (*p*-AMPK) and ULK1 at Ser555 (*p*-ULK1 Ser555), while concurrently reducing the phosphorylation of PI3K (p-PI3K Tyr458), AKT (*p*-AKT), mTOR (*p*-mTOR), ULK1 at Ser757 (*p*-ULK1 Ser757), and p70S6K (p-p70S6K) (Fig. 6A–H). These results suggest concurrent activation of the AMPK/ULK1 axis and suppression of the PI3K/AKT/mTOR signaling cascade.

To confirm the roles of AMPK and ULK1 in GPF-mediated mitophagy, cells were co-treated with GPF and either Compound C (CC, 5 ​μM), a selective AMPK inhibitor, or ULK-101 (5 ​μM), a ULK1-specific inhibitor. Fluorescence microscopy revealed that GPF-induced GFP-LC3 puncta formation was significantly attenuated in the presence of either inhibitor (Fig. 6I and J), supporting the functional involvement of these kinases in the mitophagic response. Collectively, these findings indicate that GPF activates mitophagy by stimulating the AMPK/ULK1 pathway and concurrently inhibiting the PI3K/AKT/mTOR pathway, further elucidating its mechanism of action in cellular models of AD.

### GPF promotes the autophagic degradation of AD-associated proteins in cellular models

**Fig. 7:**
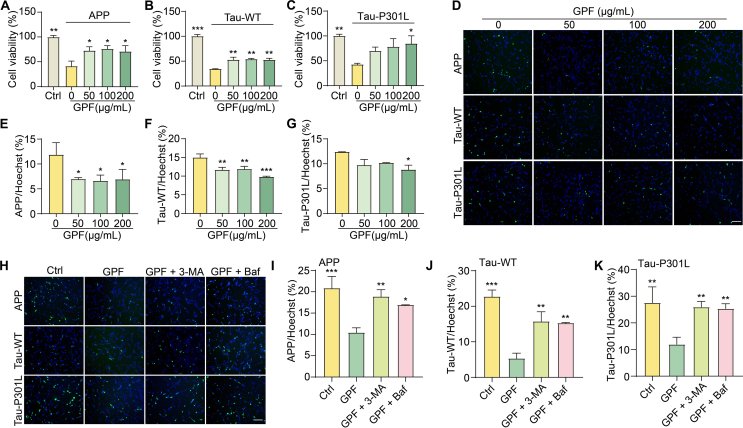
GPF promotes the autophagic degradation of AD-associated pathological proteins in PC-12 cells. (**A-C**) Cell viability of PC-12 cells transfected with pEGFP-N1-APP, pRK5-EGFP-Tau, or pRK5-EGFP-Tau-P301L and treated with GPF at 50, 100, or 200 μg/mL. Data are shown as mean ± SEM. ∗*p* < 0.05, ∗∗*p* < 0.01 vs. untreated cells. (**D**) Representative fluorescence microscopy images of PC-12 cells expressing pEGFP-N1-APP, pRK5-EGFP-Tau, or pRK5-EGFP-Tau-P301L treated with varying concentrations of GPF. Hoechst staining (blue) marks the nuclei. Magnification: × 10; scale bar: 100 μm. (**E-G**) Quantification of APP, Tau-WT, and Tau-P301L expression normalized to Hoechst staining in PC-12 cells treated with different concentrations of GPF. Data are shown as mean ± SEM. ∗*p* < 0.05, ∗∗*p* < 0.01, ∗∗∗*p* < 0.001 vs. untreated cells. (**H**) Representative fluorescence microscopy images of PC-12 cells expressing the same constructs and treated with GPF (100 μg/mL) alone or in combination with autophagy inhibitors 3-MA (5 mM) or Baf (10 μM). Hoechst staining (blue) marks the nuclei. Magnification: × 10; scale bar: 100 μm. (**I**–**K**) Quantification of APP, Tau-WT, and Tau-P301L expression normalized to Hoechst in PC-12 cells treated with GPF, 3-MA, Baf, or their combinations. Data are shown as mean ± SEM. ∗*p* < 0.05, ∗∗*p* < 0.01, ∗∗∗*p* < 0.001 vs. untreated cells.

To investigate the effects of GPF on the degradation of AD-associated pathological proteins, we conducted cell viability assays and fluorescence microscopy in PC-12 ​cells expressing pEGFP-N1-APP, pRK5-EGFP-Tau, and pRK5-EGFP-Tau-P301L. Treatment with GPF at 50, 100, and 200 ​μg/mL significantly enhanced cell viability in a dose-dependent manner, indicating protective effects against protein-induced cytotoxicity (Fig. 7A–C). Fluorescence images showed a corresponding reduction in fluorescence intensity, suggesting decreased intracellular levels of these proteins (Fig. 7D–G). To verify these effects in human neuronal cells, findings in human neuronal cells, we repeated the experiments in SH-SY5Y cells. GPF treatment significantly decreased the percentage of PI-positive cells expressing APP, Tau-WT, or Tau-P301L (Fig. S6), accompanied by a dose-dependent reduction in fluorescence intensity (Fig. S7). To determine whether these effects were mediated through autophagy, we co-treated cells with autophagy inhibitors 3-MA (5 ​mM) and Baf (10 ​μM). Both inhibitors significantly attenuated the GPF-induced degradation of APP, Tau-WT, and Tau-P301L, as shown by fluorescence images (Fig. 7H) and quantification (Fig. 7I–K), indicating that GPF promotes degradation of these proteins via an autophagy-dependent pathway. These findings support the role of GPF in enhancing autophagic clearance of AD-associated pathological proteins and mitigating their cytotoxic effects.

### GPF activates mitophagy in *C. elegans*

**Fig. 8:**
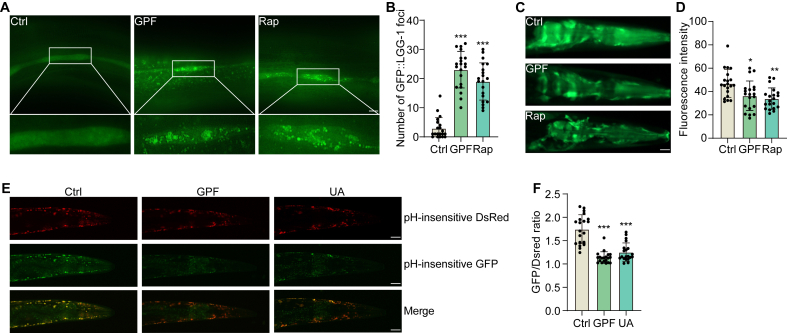
GPF activates mitophagy in *C. elegans*. (**A**) Representative fluorescence microscopy images of DA2123 worms showing GFP::LGG-1 puncta following treatment with Ctrl, GPF (100 μg/mL), or Rap (1 μM). Enlarged insets highlight increased puncta formation in the GPF- and Rap-treated groups. (**B**) Quantification of GFP::LGG-1 puncta in DA2123 worms across treatment groups. Data are expressed as mean ± SEM. ∗∗∗*p* < 0.001 vs. the Ctrl group. (**C**) Representative fluorescence images of p62:GFP expression in BC12921 worms treated with Ctrl, GPF, or Rap. (**D**) Quantification of p62:GFP fluorescence intensity in BC12921 worms. Data are presented as mean ± SEM. ∗*p* < 0.05, ∗∗*p* < 0.01 vs. the Ctrl group-. (**E**) Representative fluorescence images of IR1631 worms showing colocalization of pH-sensitive GFP and pH-insensitive DsRed signals following treatment with Ctrl, GPF, or urolithin A (UA, 50 μM). Enhanced colocalization indicates increased mitophag. (**F**) Quantification of the GFP/DsRed fluorescence ratio in IR1631 worms. Data are presented as mean ± SEM. ∗∗∗*p* < 0.001 vs. the Ctrl group.

To further validate the mitophagy-inducing properties of GPF, we employed multiple *C. elegans* models. In DA2123 transgenic worms expressing GFP::LGG-1, treatment with GPF and Rap significantly increased GFP::LGG-1 puncta formation, as observed through fluorescence microscopy and subsequent quantification, indicating enhanced autophagic activity (Fig. 8A and B). To assess the degradation of autophagic substrates, we evaluated p62 expression in BC12921 worms. GPF and Rap treatments markedly reduced p62 fluorescence intensity, suggesting accelerated autophagic flux (Fig. 8C and D). Mitophagy was further examined in IR1631 worms using a dual fluorescent reporter expressing pH-insensitive DsRed and GFP. GPF and UA treatments significantly decreased the GFP/DsRed fluorescence ratio compared to control worms, indicating increased lysosomal degradation of mitochondria (Fig. 8E and F). Taken together, these results provide in vivo evidence that GPF enhances both autophagy and mitophagy in *C. elegans*, reinforcing its mechanistic relevance and therapeutic potential.

### GPF alleviates AD-related pathology and behavioral deficits via mitophagy induction in *C. elegans*

**Fig. 9:**
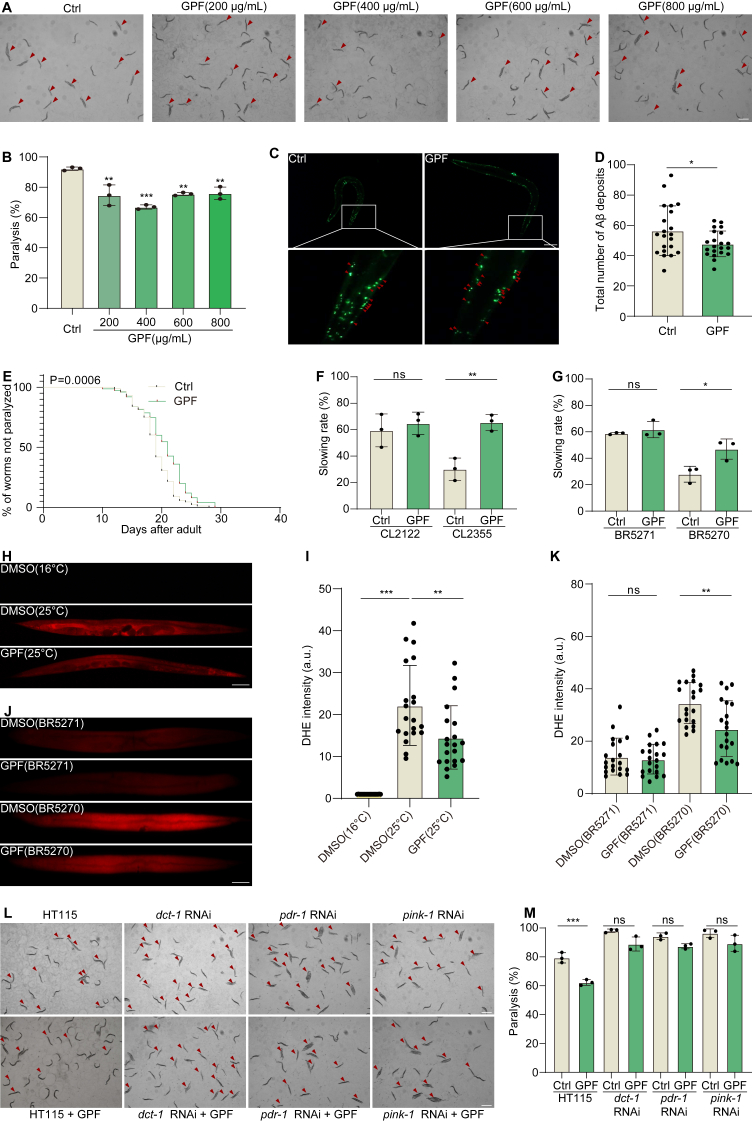
GPF alleviates AD-related pathology and behavioral deficits via mitophagy induction in *C. elegans*. (**A**, **B**) Representative images and quantification of paralysis rates in CL4176 worms treated with various concentrations of GPF (200, 400, 600, and 800 μg/mL). Red arrows indicate non-paralyzed worms. Magnification: 20 × ; scale bar: 1 mm. Data are presented as means ± SEM. ∗∗*p* < 0.01, ∗∗∗*p* < 0.001 vs. the Ctrl group. (**C**, **D**) Representative fluorescence microscopy images and quantification of Aβ(3–42) deposits in CL2331 worms treated with GPF (800 μg/mL). Red arrows indicate visible Aβ deposits. Data are presented as mean ± SEM. ∗*p* < 0.05 vs. the Ctrl group. (**E**) Kaplan‒Meier survival curves showing the percentage of non-paralyzed CL2006 worms over time following treatment with Ctrl or GPF (400 μg/mL). Significance was determined by the log-rank test. (**F**, **G**) Quantification of food-sensing behavior (slowing rate) in CL2122 and CL2355 worms treated with Ctrl or GPF (400 μg/mL). Data are shown as mean ± SEM. ∗*p* < 0.05, ∗∗*p* < 0.01 vs. the Ctrl group. (**H**–**K**) Representative fluorescence microscopy images and quantification of DHE fluorescence intensity to assess reactive oxygen species (ROS) levels in CL4176 worms (at 16 °C and 25 °C) and in BR5270 and BR5271 worms treated with DMSO or GPF. Magnification: 10 × ; scale bar: 100 μm. Data are presented as means ± SEM. ∗∗*p* < 0.01, ∗∗∗*p* < 0.001 vs. the DMSO Ctrl group. (**L**, **M**) Representative images and quantification of paralysis rates in CL4176 worms treated with GPF and subjected to RNAi-mediated knockdown of *dct-1*, *pdr-1*, or *pink-1*. RNAi abrogated the protective effect of GPF. ∗∗∗*p* < 0.001 vs. the Ctrl group.

To evaluate the therapeutic potential of GPF in *C. elegans* models of AD, we first assessed its effect on paralysisin CL4176 worms, which express muscle-specific Aβ1‒42 under a temperature-inducible promoter. Upon temperature upshift from 16 ​°C to 25 ​°C, treatment with GPF (200–800 ​μg/mL) significantly delayed paralysis in a dose-dependent manner, with 400 ​μg/mL GPF yielding the most significant improvement (Fig. 9A and B). To investigate GPF's effect on Aβ aggregation, we employed CL2331 woems expressing GFP-tagged Aβ3-42 in body wall muscle cells. GPF treatment significantly reduced the number of GFP-positive puncta, indicating diminished Aβ deposits (Fig. 9C and D). In CL2006 worms, which constitutively express Aβ3-42, GPF treatment similarly delayed the paralysis (Fig. 9E). We next examined behavioral phenotypes related to sensory perception. In CL2355 worms, which display Aβ-induced deficits in food perception, GPF significantly increased the food-slowing response compared to untreated animals (Fig. 9F). A similar effect was observed in BR5270 worms, which express human tau panneuronally, and exhibit tau-induced deficits in food perception. GPF treatment restored food-slowing behavior, comparable to control strain BR5271 (Fig. 9G). To assess antioxidant capacity, we measured ROS levels in Aβ- and tau-expressing worms using DHE staining. GPF treatment markedly reduced DHE fluorescence, indicating a reduction in oxidative stress (Fig. 9H–K). To investigate whether the protective effects of GPF are mitophagy-dependent, we knocked down key mitophagy-related genes (*dct-1*, *pdr-1*, and *pink-1*) in CL4176 worms using RNAi [35]. GPF significantly delayed paralysis in worms fed control HT115 bacteria, but this effect was abolished upon RNAi-mediated suppression of mitophagy genes (Fig. 9L and M). Taken together, these results indicate that GPF mitigates AD-like pathology and behavioral deficits in *C. elegans* models by enhancing mitophagy, reducing Aβ and tau toxicity, and alleviating oxidative stress.

## Discussion

AD is a devastating neurodegenerative disorder affecting millions of people worldwide [41]. Although the exact cause of AD remains unknown, researchers have identified misfolded proteins, including Aβ plaques and neurofibrillary tangles (NFTs), as two hallmarks of AD [42]. Aβ plaques form extracellularly, and NFTs form intracellularly, disrupting communication between neurons, impairing brain function, and ultimately leading to neuronal death [43]. Autophagy is responsible for the degradation and recycling of damaged organelles, long-lived proteins, and other types of cellular waste [44]. This process is particularly important in neurons, as they have a limited capacity for self-renewal and rely heavily on autophagy to maintain optimal function [45]. However, recent studies have shown that autophagy impairment is linked to the accumulation of both Aβ plaques and Tau tangles, suggesting a critical role in the pathogenesis of AD [46]. Our study introduces several novel elements to the field of AD research, contributing to ongoing efforts to develop more effective therapeutic strategies for this complex neurodegenerative disorder. First, our dual-targeting approach, which simultaneously addresses Aβ fibrillization and enhances mitophagy, represents an innovative strategy that may offer more comprehensive neuroprotection than traditional single-target approaches. This multifaceted approach is particularly important in the context of AD, where the complex pathology involves multiple interrelated processes. By targeting both the accumulation of toxic Aβ fibrils and the associated cellular clearance mechanisms, we aim to address two critical aspects of AD pathogenesis concurrently. This strategy may yield synergistic effects, enhancing overall neuroprotection beyond what might be achieved by targeting either process alone. Moreover, this approach aligns with the growing recognition in the field that combination therapies may be necessary to effectively combat the multifactorial nature of AD. Therefore, we now shift our focus to dual-targeting strategies that concurrently address both Aβ fibrillization and the autophagic degradation of pathogenic proteins, potentially providing a more comprehensive and efficacious treatment for AD [47].

The use of natural medicines derived from plants, animals, and microorganisms has a longstanding history in traditional medicine, especially in countries such as China and Japan. With the progression of modern science, researchers have commenced probing the potential of these natural resources for drug screening and discovery because of their advantageous efficacy, diversity, sustainability, and reduced propensity for resistance development [48,49]. In this study, methanol was employed as the extraction solvent to extract these natural herbs because of its superior ability to extract a broad spectrum of bioactive compounds. We recognize that its use is not suitable for public health applications, particularly because of its toxicity. Therefore, in future studies, we will optimize the extraction process using safer solvents such as ethanol, which is more appropriate for potential therapeutic applications. Through extensive screening of our established natural medicine library via the ThT assay, followed by cytotoxicity examination in Aβ-induced PC-12 ​cells, we verified the efficacy of 15 natural medicine extracts in inhibiting Aβ fibrillization. We subsequently determined the autophagy activity of these 12 extracts in stable RFP-GFP-LC3 U87 ​cells and PC-12 ​cells and discovered that extract A7, derived from the entirety of *G. leucocarpa*, could activate autophagy. As a result, we selected *G. leucocarpa* extract (GE) for subsequent experiments, including rudimentary fractionation, chemical characterization, assessment of anti-AD efficacy, and exploration of the mechanism of action of the GPF obtained from GE. Therefore, our exploration of *G. leucocarpa* complements this dual-targeting approach by introducing a novel plant species to the realm of AD research. This plant, which was not previously investigated in the context of AD, expands the repertoire of potential natural therapeutic agents. The rich phytochemical profile of *G. leucocarpa*, which includes various flavonoids, phenolic acids, and terpenoids, offers a promising source of bioactive compounds with potential neuroprotective properties. By identifying new natural sources of therapeutic agents, we contribute to the diversification of drug discovery efforts in AD research, potentially uncovering compounds with unique mechanisms of action or improved efficacy compared with existing options. Furthermore, our study bridges the gap between traditional herbal medicine and cutting-edge neuroscience, potentially opening new avenues for drug discovery in neurodegenerative diseases. This integration of traditional knowledge with modern scientific approaches represents a valuable synergy in medical research. By subjecting traditional herbal remedies to rigorous scientific investigation, we can potentially uncover new therapeutic agents while also providing a scientific basis for traditional practices. This approach not only honors the historical contributions of traditional medicine but also leverages centuries of empirical observations to guide modern drug discovery efforts. In the context of AD, where current treatments offer limited efficacy, this integration of traditional and modern approaches could lead to breakthrough discoveries.

Natural medicines offer a vast array of structurally diverse and bioactive compounds. This chemical diversity proves advantageous for drug discovery, as it increases the likelihood of identifying novel compounds with unique mechanisms of action. In this study, the preliminary separation of the components in GE was executed based on the distinct polarity of the chemicals, resulting in a total of four fractions, including GWF, GNF, GEF, and GPF. In the subsequent validation of the effects of these fractions on the inhibition of Aβ fibrillization and autophagy activation, we found that all four fractions inhibited Aβ fibrillization at various time points and reduced cell death in Aβ1-42-induced PC-12 ​cells. However, only GPF and GEF significantly increased GFP-LC3 puncta formation, with GPF exhibiting a superior effect. This may be due to the presence of components with autophagy induction potential in GPF and GEF, and a relatively high ratio in GPF. Consequently, GPF can not only inhibit Aβ fibrillization but also activate autophagy in PC-12 ​cells. A UHPLC-Q/TOF-MS instrument was subsequently employed to perform chemical identification, revealing the presence of several characteristic components, such as gaultherin, gaultherin A, and gaultherin B, in the GPF. Although the specific components in GPF responsible for Aβ fibrillization inhibition and autophagy activation remain undetermined after the initial separation of GE, this information still offers valuable insights for further chemical isolation and identification, with future studies focusing on isolating and identifying such components from GPF.

**Fig. 10:**
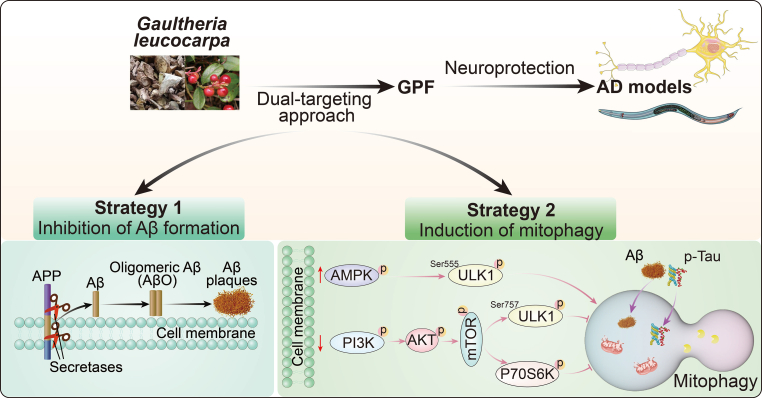
Schematic diagram of this study. This schematic illustrates the dual-targeting strategy employed in this study to identify GPF from *G. leucocarpa* as a therapeutic candidate for AD. GPF concurrently inhibits Aβ fibrillization and enhances mitophagic degradation of pathogenic proteins in both cellular and C. *elegans* models of AD. Mechanistically, GPF promotes the clearance of Aβ fibrils and p-Tau by activating mitophagy through the AMPK/ULK1 and PI3K/AKT/mTOR signaling pathways.

In subsequent experiments, our molecular target analysis provide significant insights into the potential mechanisms through which GPF exerts its neuroprotective effects, particularly in the context of AD. The identification of 40 shared genes between GPF targets and AD-related genes emphasizes the potential of GPF compounds to modulate key AD-related biological processes. The enrichment of these genes in pathways such as Aβ metabolism and apoptosis is particularly noteworthy, as it aligns with previous experimental findings demonstrating that GPF inhibits Aβ protein formation. This suggests that GPF could target the pathological buildup of Aβ, possibly preventing or slowing disease progression. Moreover, the enrichment of the PI3K-AKT signaling pathway and autophagy-related pathways underscores the potential role of GPF in modulating cellular survival and degradation processes, both of which are critical in AD pathology. PI3K-AKT signaling is known to play a protective role in neurodegenerative diseases by promoting cell survival, while autophagy is crucial for clearing damaged proteins and organelles, such as Aβ and defective mitochondria. Thus, the modulation of these pathways by GPF may contribute to its therapeutic potential in AD. These findings not only support the hypothesis that GPF can inhibit Aβ formation but also suggest that it may have broader neuroprotective effects by regulating oxidative stress, mitochondrial function, apoptosis, and autophagy. Further experimental validation was performed to confirm these potential mechanisms and to explore the therapeutic efficacy of GPF in cellular and worm models of AD. mTOR is a serine/threonine kinase that functions as a central regulator of cell growth, proliferation, and survival [50]. mTOR exists in two distinct complexes: mTOR complex 1 (mTORC1) and mTOR complex 2 (mTORC2) [51]. The mTORC1 pathway is the primary regulator of autophagy, and its activity is influenced by various factors, such as nutrient availability, growth factors, and the cellular energy status. AMPK is a central energy-sensing enzyme that monitors the cellular AMP/ATP ratio. Under energy-depleted conditions, such as glucose starvation, the AMP/ATP ratio increases, leading to AMPK activation [52]. Mitophagy activation by AMPK occurs via direct phosphorylation of several autophagy-related proteins, such as ULK1 and Beclin-1, and indirectly through inhibition of mTORC1 activity [53]. Therefore, the dynamic balance between AMPK and mTOR signaling is crucial for the regulation of autophagy/mitophagy. In this study, Western blotting was used to detect the protein expression of AMPK-mTOR signaling-related proteins, revealing that GPF significantly increased the phosphorylation of AMPK and decreased the phosphorylation of PI3K, AKT, mTOR, and P70s6K. Concurrently, treatment with ULK-101 (ULK1 inhibitor) and CC (AMPK inhibitor) reversed the effect of GPF on mitophagy activation. Therefore, these results demonstrate that AMPK‒mTOR signaling participates in the regulation of GPF-induced mitophagy. Furthermore, in PC-12 ​cells transiently transfected with pEGFP-N1-APP, pRK5-EGFP-Tau, or pRK5-EGFP-Tau-P301L, GPF reduced the expression of GFP and increased the viability of PC-12 ​cells, which was reversed by the application of 3-MA and CC. These results suggest that GPF promotes the degradation of AD-related proteins via AMPK-mTOR-mediated autophagy (Fig. 10). Our focus on mitophagy enhancement as a therapeutic strategy in AD highlights an area that has been relatively underexplored compared with direct targeting of amyloid pathology. While much AD research has focused on Aβ and tau pathology, the role of mitochondrial dysfunction and impaired cellular clearance mechanisms has gained increasing attention in recent years. By emphasizing mitophagy enhancement, our study contributes to this emerging area of research, potentially opening new therapeutic avenues. In this study, while our study utilized PC-12 ​cells, we recognize the importance of validating our findings in primary hippocampal neurons. Future studies will aim to replicate these results in primary neuronal cultures to further elucidate the neuroprotective effects of GPF in a more physiologically relevant context.

*C. elegans* is a widely used model organism in biological research because of its simplicity, short lifespan, and ease of genetic manipulation. Its gene has high homology with that of humans, so it is often used to study human aging and neurological diseases [54,55]*.* In recent years*, C. elegans* has emerged as a powerful tool for investigating the molecular mechanisms underlying autophagy and AD [38]. In this study, we utilized the DA2123 and BC12921 strains to determine the expression of LGG-1 and p62 in nematodes. Our findings corroborate that GPF activates autophagy in worms, and the diminished ratio of pH-sensitive GFP to pH-insensitive DsRed in GPF-treated IR1631 worms suggests that GPF induces mitophagy. Although the autophagy induction of GPF in worms was verified in this study, the regulatory mechanism of autophagy remains unconfirmed. In subsequent research, isolated compounds that induce autophagy will be employed to explore the mechanism of action in *C. elegans*. To evaluate the anti-AD effect of the GPF, we utilized Aβ and tau transgenic worms to examine the ameliorative effect of the GPF on AD-related behavior and pathology. The results demonstrated that GPF ameliorated Aβ-induced short-term and long-term paralysis, as well as Aβ- or tau-induced deficits in food-sensing behavior. Additionally, GPF diminished Aβ deposits in CL2331 worms and decreased ROS levels in Aβ and tau transgenic worms, CL4176 and BR5270. Nevertheless, feeding with RNAi *dct-1*, RNAi *pdr-1*, or RNAi *pink-1* bacteria counteracted the ameliorative effect of GPF on paralysis in CL4176 worms. These findings suggest that GPF exerts a neuroprotective effect via the induction of autophagy/mitophagy. While the current study provides valuable insights into the potential of GPF in AD treatment, we acknowledge that more advanced AD animal models, such as APP/PS1 mice and 3 ​× ​AD mice, would offer a more robust platform for exploring the effects and mechanisms of our compounds [34,56]. As we progress from extract-level to ingredient-level investigations, our future studies will utilize these models to further validate our findings and gain deeper insights into the therapeutic potential of the active ingredients of *G. leucocarpa* in AD treatment.

A key limitation of the current study is the lack of identified specific pharmacological targets for GPF. While we observed significant effects on Aβ fibrillization and mitophagy, the precise molecular interactions underlying these effects remain to be elucidated. Based on our observations, we hypothesize that components of GPF may interact with proteins involved in Aβ aggregation (such as BACE1 or γ-secretase) and/or mitophagy regulation (such as PINK1, Parkin, or OPTN). Future studies will focus on validating these potential targets through molecular docking simulations and *in vitr*o binding assays. Our ongoing and future work will focus on isolating active compounds from GPF and conducting comprehensive target identification studies. These methods include in silico molecular docking, *in vitro* binding assays, and proteomics approaches such as drug affinity responsive target stability (DARTS) and cellular thermal shift assays (CETSAs). These studies provide crucial insights into the specific molecular mechanisms by which GPF affects Aβ fibrillization and mitophagy.

Collectively, these aspects of our study point toward the potential for developing multimodal therapeutic approaches based on natural products, which could prove more effective in addressing the complex pathology of AD. The multitarget effects observed with GPF suggest that natural products may offer inherent advantages in treating complex diseases such as AD. Unlike single-compound drugs designed to interact with specific targets, natural products often contain a variety of bioactive compounds that may act on multiple pathways simultaneously. This multimodal action could be particularly beneficial in AD, where the interplay of various pathological processes contributes to disease progression. This integrated approach not only offers new insights into potential AD treatments but also establishes a framework for future studies exploring multifaceted, nature-inspired interventions in neurodegenerative diseases such as AD. By demonstrating the potential of combining traditional herbal knowledge with modern neuroscience techniques and multitarget strategies, our study paves the way for similar approaches in investigating other neurodegenerative disorders. This framework could inspire researchers to explore other understudied natural products, investigate multitarget approaches, and integrate traditional medicine with cutting-edge scientific methodologies. In conclusion, this study presents a novel dual-targeting approach using GPF for AD treatment, innovatively combining Aβfibrillization inhibition with mitophagy enhancement via the AMPK‒mTOR signaling pathway. This study not only introduces a new potential therapeutic agent but also establishes a framework for future studies exploring multimodal, natural product-based interventions in neurodegenerative diseases such as AD. Moreover, our ongoing work focuses on isolating the active ingredients from GPF. Future studies will involve a systematic activity evaluation of these isolated compounds using advanced AD models, including APP/PS1 and 3 ​× ​AD mice. These investigations will allow us to better understand the specific effects of each active ingredient and to further elucidate their molecular targets and mechanisms of action in the context of AD pathology.

## Ethics approval and consent to participate

Not applicable.

## Data availability

The data that support the findings of this study are available from the corresponding author upon reasonable request.

## Author contributions

**Yue Zhang:** Data curation, Methodology. **Lan Deng:** Data curation, Methodology. **Jing Wei:** Data curation. **Lufen Huang:** Investigation, Validation. **Fei Gao:** Data curation. **Lu Yu:** Investigation, Validation. **Fengdan Zhu:** Data curation. **Jianing Mi:** Investigation. **Jianming Wu:** Writing – review & editing. **Fang Ren:** Writing – review & editing. **Minsong Guo:** Data curation. **Xiaogang Zhou:** Funding acquisition, Supervision. **Dalian Qin:** Conceptualization, Funding acquisition, Supervision. **Ting Chen:** Funding acquisition, Writing – review & editing. **Anguo Wu:** Supervision, Writing – original draft, Writing – review & editing.

## Declaration of competing interest

The authors declare no conflicts of interest.

## Footnotes

Appendix A Supplementary data to this article can be found online at https://doi.org/10.1016/j.neurot.2025.e00721.
