# Supplementary material for: Gaultheria leucocarpa inhibits Aβ fibrillization and enhances mitophagy-mediated degradation of pathogenic proteins
Source: Neurotherapeutics. 2025 Aug 14;22(6):e00721. doi: 10.1016/j.neurot.2025.e00721 (PMC12664514; doi:10.1016/j.neurot.2025.e00721)
Supplement: Multimedia component 1 [file mmc1.docx]

**Supplementary materials**

***Gaultheria leucocarpa* inhibits Aβ fibrillization and enhances mitophagy-mediated degradation of pathogenic proteins**

Yue Zhang^a,†^, Lan Deng^a,†^, Jing Wei ^a,†^, Lufen Huang^c,†^, Fei Gao ^a^, Lu Yu^a^, Fengdan Zhu^a^, Jianing Mi^d^, Jianming Wu^a^, Fang Ren^e^, Minsong Guo^a^, Xiaogang Zhou^a,^*, Dalian Qin^a,^*, Ting Chen^b,^*, Anguo Wu^a,d^*

*^a^Luzhou Key Laboratory of Activity Screening and Druggability Evaluation for Chinese Materia Medica, Key Laboratory of Medical Electrophysiology of Ministry of Education, Department of Cardiology, Department of Ophthalmology, The Affiliated Hospital of Southwest Medical University, School of Pharmacy, Southwest Medical University, Luzhou, China, 646000. zhangyue9680@163.com (Y. Z.); denglan910@163.com (L. D.); weijingswmu@163.com (J. W.); gaofei202403@163.com (F. G.); yulu863@swmu.edu.cn (L. Y.); lethe19970801@163.com (F. Z.);* *jianmingwu@swmu.edu.cn (J. W.); dididigms@163.com (M. G.); zxg@swmu.edu.cn (X. Z.); dalianqin@swmu.edu.cn (D. Q.); wuanguo@swmu.edu.cn (A. W.).*

*^b^School of Pharmaceutical Sciences, China-Pakistan International Science and Technology Innovation Cooperation Base for Ethnic Medicine Development in Hunan Province, Hunan University of Medicine, Huaihua, China, 418000.* *chenting@hnmu.edu.cn (T. C.).*

*^c^Department of Pharmacy, Jining Medical University, Rizhao 276500, China; huanglufen0029@126.com (L. H.).*

*^d^State Key Laboratory of Traditional Chinese Medicine Syndrome, The Second Affiliated Hospital of Guangzhou University of Chinese Medicine, Guangzhou, Guangdong, China, 510120. mjnrhw@hotmail.com (J. M); wuanguo@swmu.edu.cn (A. W.).*

*^e^Chongqing Key Laboratory of Sichuan-Chongqing Co-construction for Diagnosis and Treatment of Infectious Diseases Integrated Traditional Chinese and Western Medicine, Chongqing Traditional Chinese Medicine Hospital, Chongqing, 400021, China, renifang1993@163.com (F. R.).*

*Corresponding authors: Xiaogang Zhou (Tel.: +86 15908116198; E-mail: zxg@swmu.edu.cn); Dalian Qin (Tel.: +86 13518376093; E-mail: dalianqin@swmu.edu.cn); Ting Chen (Tel.: +86 07452382877; E-mail: *chenting@hnmu.edu.cn*); Anguo Wu (Tel.: +86 17769617417; E-mail: wuanguo@swmu.edu.cn)

^†^ Authors contribute to equal works.


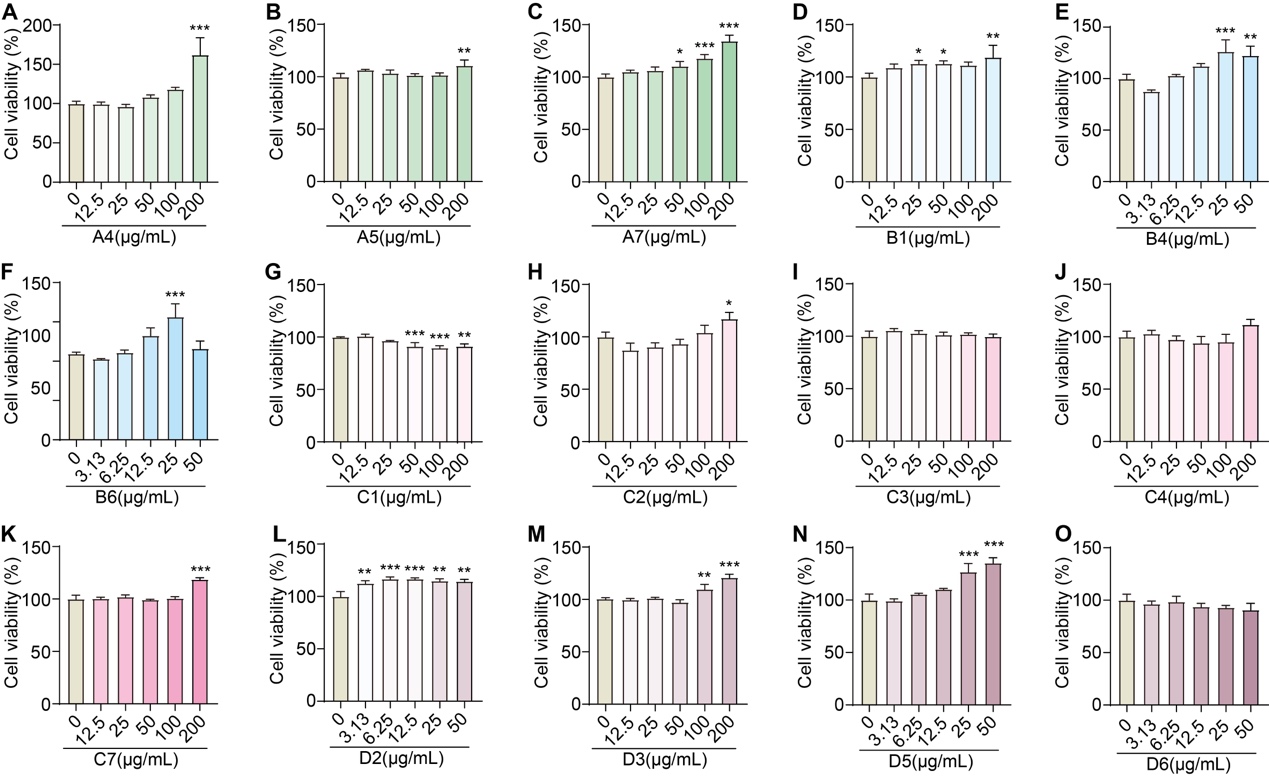


**Figure S1** Evaluation of cell viability in response to various herbal extracts. Cell viability assays were conducted to assess the cytotoxicity of different concentrations of herbal extracts on PC-12 cells. Cells were treated with extracts A4, A5, A7, B1, B4, B6, C1, C2, C3, C4, C7, D2, D3, D5, and D6 at concentrations ranging from 0 to 200 µg/mL. Cell viability was measured using a standard MTT assay. Data are presented as mean ± SEM. **p* < 0.05, ***p* < 0.01, ****p* < 0.001 vs. the Ctrl group (0 µg/mL).


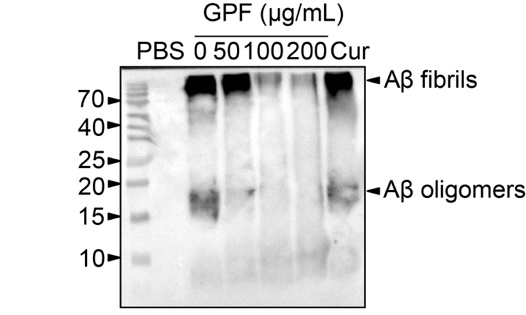


**Figure S2** GPF inhibits Aβ fibrillization and oligomerization. Western blot analysis of Aβ species in the presence of different concentrations of GPF and Cur as a positive Ctrl.


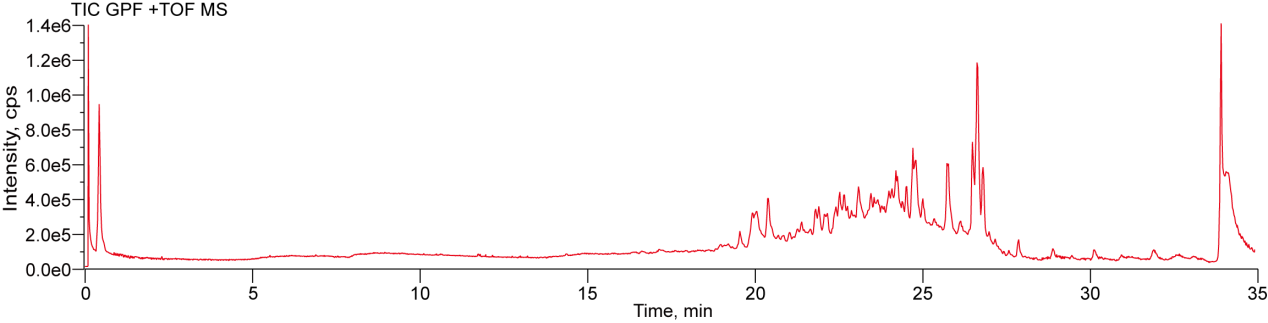


**Figure S3** The total ion chromatogram (TIC) of GPF analyzed by UHPLC-DAD-Q/TOF-MS/MS in positive ion mode.

**
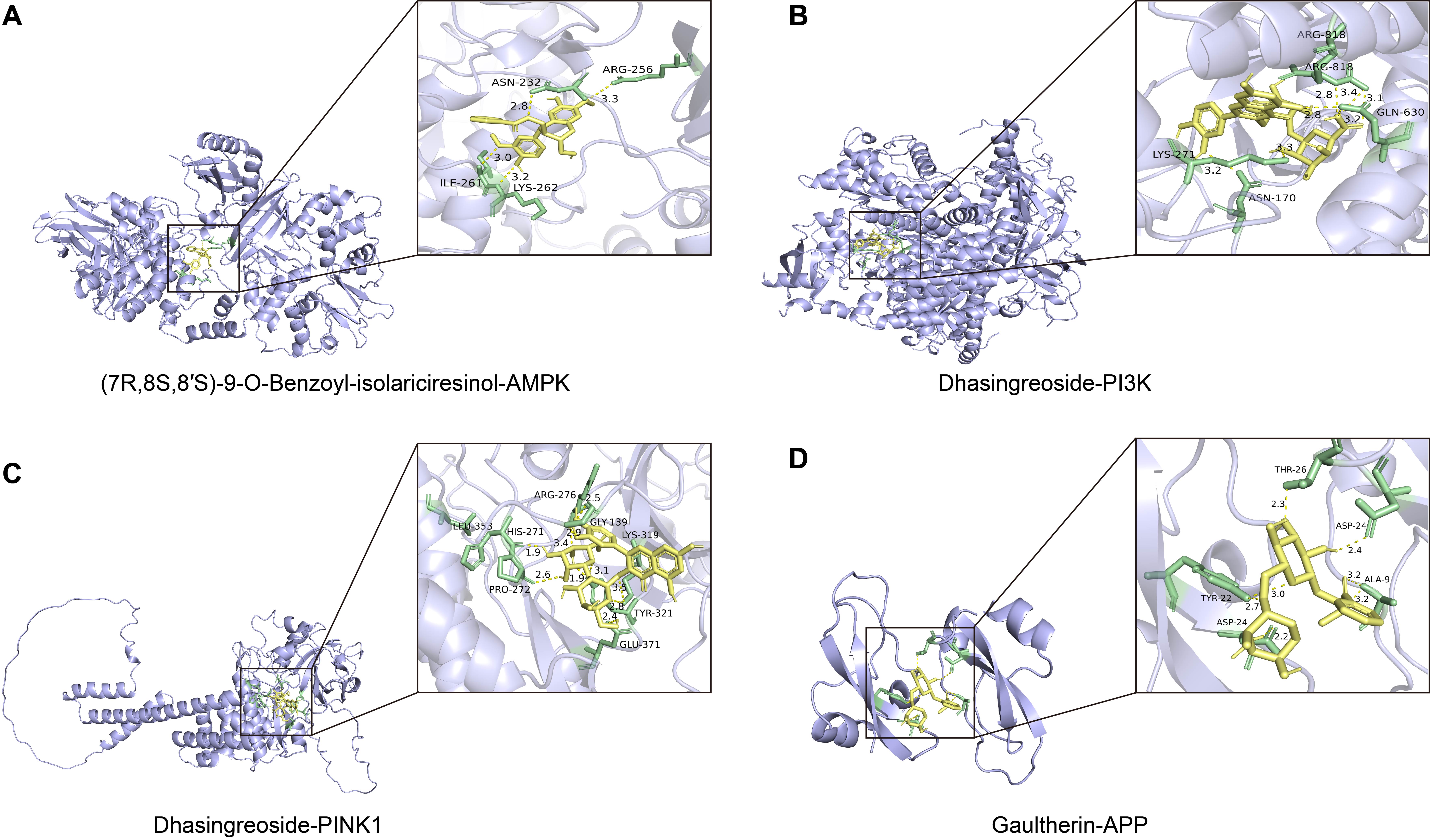
**

**Figure S4** Representative molecular docking analysis of compounds in GPF with target proteins. (A) Binding mode of (7R,8S,8’S)-9-O-Benzoyl-isolariciresinol with AMPK (PDB: 6C9H). Key amino acid residues involved in hydrogen bonding or hydrophobic interactions include ASN-232, ARG-256, ILE-261, and LYS-262. (B) Binding mode of dhasingreoside with PI3K (PDB: 4JPS). The ligand interacts with residues ARG-828, LYS-771, ASN-170, and GLN-630, forming multiple hydrogen bonds and hydrophobic contacts. (C) Binding mode of dhasingreoside with PINK1 (PDB: AF-Q9BXM7-F1). The compound is tightly docked within the binding pocket, forming interactions with ARG-276, HIS-271, LYS-319, TYR-321, and GLU-371. (D) Binding mode of gaultherin with APP (PDB: 1AAP). The compound interacts via hydrogen bonding with residues TYR-22, ASP-24, ALA-9, and THR-26, indicating potential affinity toward APP. Each inset shows the enlarged view of the ligand-binding pocket, with key residues labeled and distances (in Å) between atoms forming hydrogen bonds or other interactions indicated.


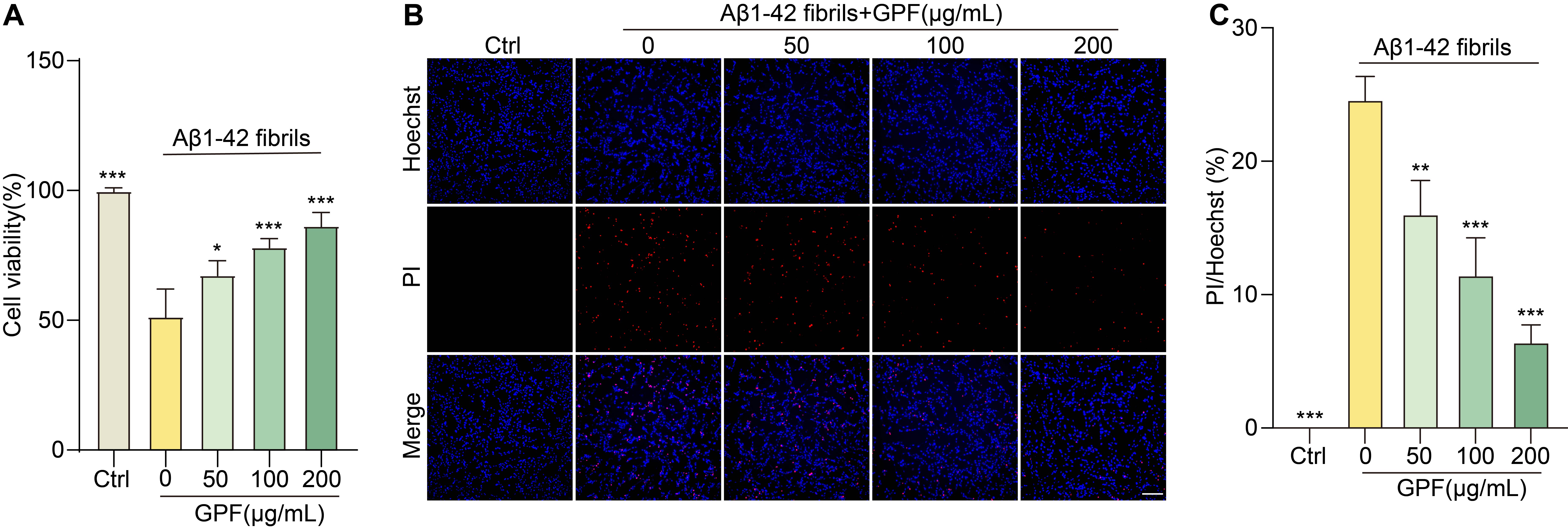


**Figure S5** GPF attenuates Aβ1–42 fibril-induced cytotoxicity in SH-SY5Y cells. (A) Cell viability was assessed using the MTT assay after SH-SY5Y cells were treated with Aβ1–42 fibrils (20 μM) in the absence or presence of GPF at concentrations of 50, 100, and 200 μg/mL for 24 h. (B) Representative fluorescence images of SH-SY5Y cells stained with Hoechst (blue) and propidium iodide (PI, red) after the indicated treatments. Magnification: ×10, Scale bar: 100 μm. (C) Quantification of PI-positive cells relative to total Hoechst-stained nuclei. Data are expressed as mean ± SEM. **p* < 0.05, ****p* < 0.001 vs. Aβ1–42 fibril-treated group.


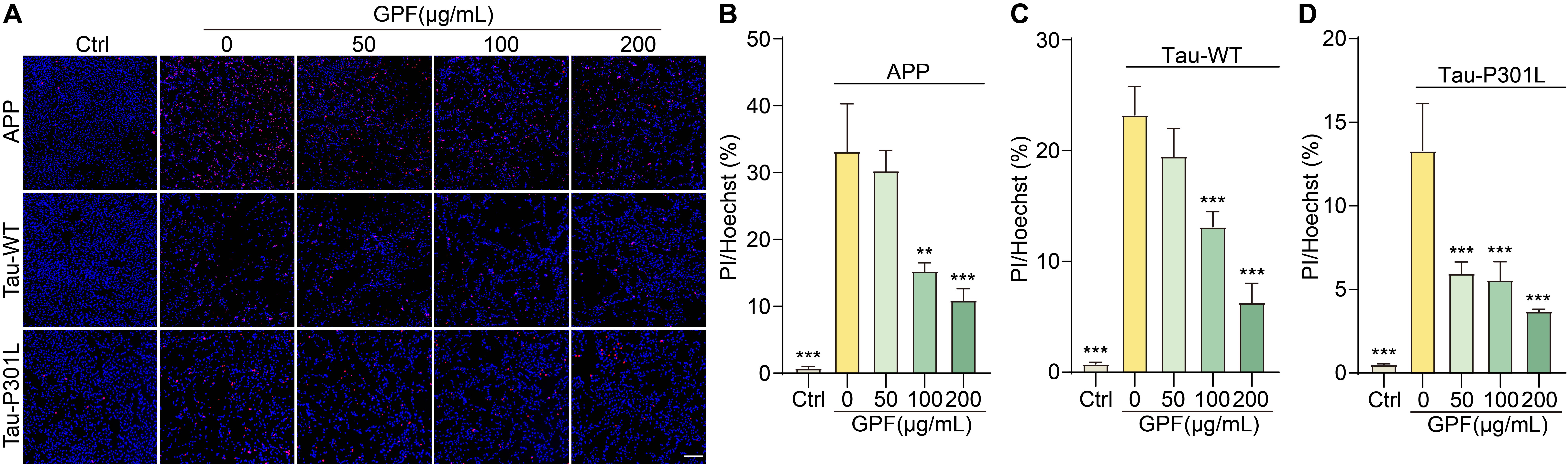


**Figure S6** GPF reduces the cytotoxicity in SH-SY5Y cells overexpressing APP, Tau-WT, or Tau-P301L. (A) Representative images of SH-SY5Y cells transfected with pEGFP-N1-APP, pRK5-EGFP-Tau, and pRK5-EGFP-Tau-P301L, stained with Hoechst (blue) and PI (red) after treatment with GPF (50, 100, 200 μg/mL) for 24 h. Magnification: ×10, Scale bar: 100 μm. (B–D) Quantification of PI-positive nuclei relative to total Hoechst-stained cells in pEGFP-N1-APP- (B), pRK5-EGFP-Tau- (C), and pRK5-EGFP-Tau-P301L-overexpressing cells (D). Data are shown as mean ± SEM (n = 3). ***p* < 0.01, ****p* < 0.001 vs. APP, Tau-WT, and Tau-P301L overexpressing groups.


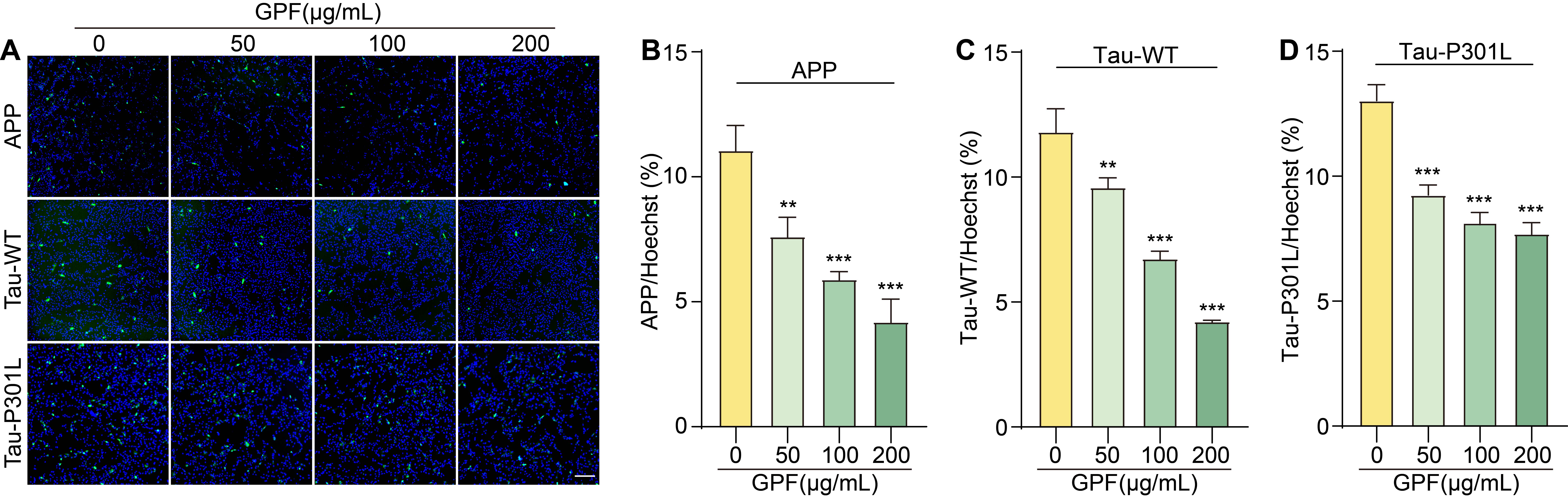


**Figure S7** GPF reduces intracellular expression of APP, Tau-WT, and Tau-P301L in SH-SY5Y cells. (A) Representative immunofluorescence images showing the expression of APP, Tau-WT, and Tau-P301L (green) in SH-SY5Y cells transfected with pEGFP-N1-APP, pRK5-EGFP-Tau, or pRK5-EGFP-Tau-P301L, and treated with increasing concentrations of GPF (50, 100, and 200 μg/mL) for 24 hours. Nuclei were stained with Hoechst (blue). Magnification: ×10, Scale bar: 100 μm. (B–D) Quantification of the fluorescence intensity ratio of APP/Hoechst (B), Tau-WT/Hoechst (C), and Tau-P301L/Hoechst (D). Data are presented as mean ± SEM. ***p* < 0.01, ****p* < 0.001 vs. the Ctrl group (0 µg/mL).


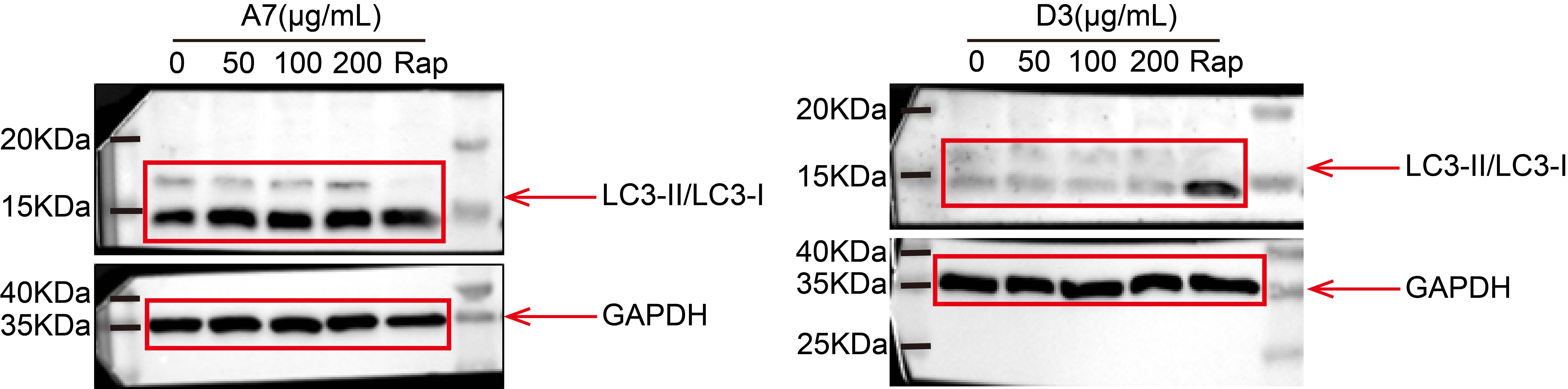


**
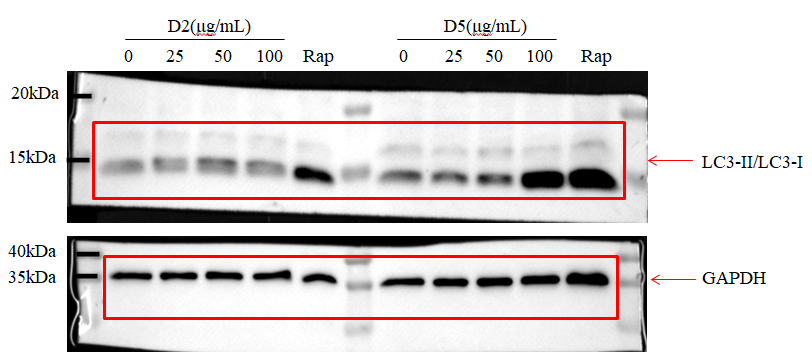
**

**
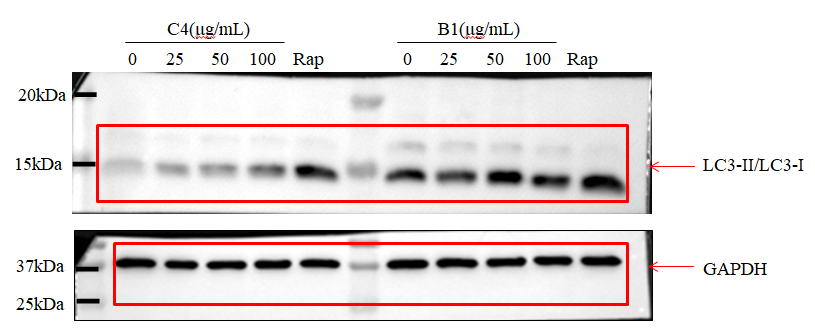
**

**Figure S8** Original and non-processed Western blot images of Figure 2 E-G and K-M.

**
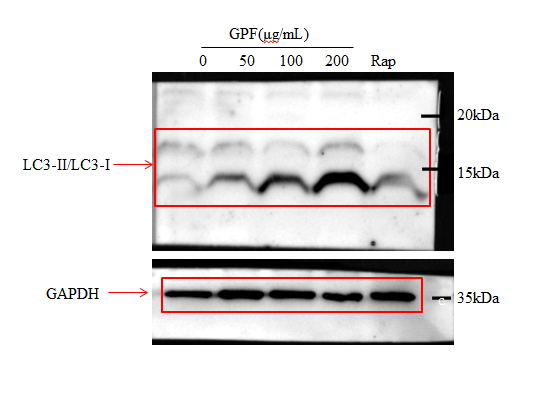
**

**Figure S9** Original and non-processed Western blot images of Figure 4C.

**
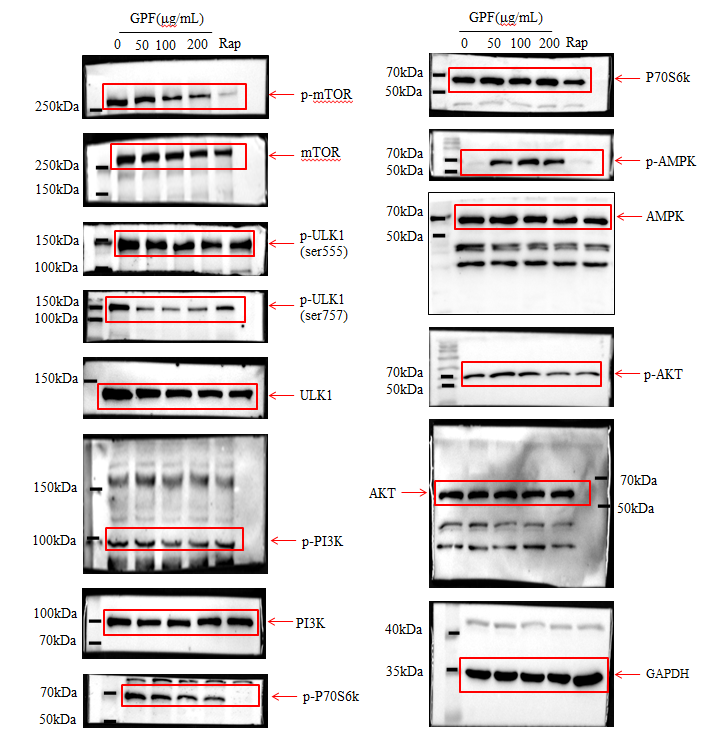
**

**Figure S10** Original and non-processed Western blot images of Figure 6A.

**Table S1**. Yields of the herbal extracts used in this study.

| **Natural herbs** | **Raw medicinal materials (g)** | **Extract (g)** | **Yield (%)** |
| --- | --- | --- | --- |
| *Achyranthes bidentata Blume* (A1) | 500 | 95.40 | 19.08 |
| *Adiantum capillus-veneris* (A2) | 500 | 123.32 | 24.66 |
| *Cucubalus baccifer* L (A3) | 500 | 95.52 | 19.10 |
| *Melastoma dodecandrum Lour* (A4) | 500 | 80.05 | 16.01 |
| *Lygodium japonicum* (Thunb.) Sw (A5) | 500 | 74.21 | 14.84 |
| Nephrolepis auriculata (A6) | 500 | 82.27 | 16.45 |
| *Gaultheria leucocarpa Bl. var. crenulata* (Kurz) T. Z (A7) | 500 | 91.86 | 18.37 |
| *Munronia henryi Harms* (A8) | 500 | 89.79 | 17.96 |
| *Toddalia asiatica* (L.) Lam (B1) | 500 | 107.21 | 21.44 |
| *Camellia reticulata Lindl* (B2) | 500 | 80.30 | 16.06 |
| *Lycopodium serratum Thunb* (B3) | 500 | 78.66 | 15.73 |
| Lysionotus serratus D. Don(B4) | 500 | 112.33 | 22.47 |
| *PeperomiadindygulensisMiq* (B5) | 500 | 87.34 | 17.47 |
| *Galium elegans Wall. ex* Roxb (B6) | 500 | 71.90 | 14.38 |
| *Pueraria lobata* (B7) | 500 | 117.43 | 23.49 |
| *Ficus tikouaBur* (B8) | 500 | 99.22 | 19.84 |
| *Valeriana jatamansi* Jones (C1) | 500 | 95.43 | 19.09 |
| *Dysosma versipellis* (C2) | 500 | 80.20 | 16.04 |
| *Gentiana rhodantha* (C3) | 500 | 106.77 | 21.35 |
| *Hypericum japonicum Thunb* (C4) | 500 | 86.24 | 17.25 |
| *Nothopanax davidii Franch.Harms* (C5) | 500 | 91.33 | 18.27 |
| *Achyranthes longifolia* (Makino) Makino (C6) | 500 | 84.76 | 16.95 |
| *Puerariae Radix* (C7) | 500 | 93.47 | 18.69 |
| *EpimediumbrevicornuMaxim.* (D1) | 500 | 100.01 | 20.00 |
| *Kummerowia striata (Thunb.) Schindl* (D2) | 500 | 98.40 | 19.68 |
| *Korthalsella japonica(Thunb.) Engl* (D3) | 500 | 88.96 | 17.79 |
| *Myosotonaquaticum(L.) Moench* (D4) | 500 | 104.20 | 20.84 |
| *Periploca forrestii Schltr* (D5) | 500 | 89.90 | 17.98 |
| *Potentilla kleiniana Wight et Arn* (D6) | 500 | 78.43 | 15.69 |
| *Ainsliaea pertyoides Franch. Var* (E1) | 500 | 121.30 | 24.26 |
| *Arisaema erubescens (Wall.) Schott* (E2) | 500 | 84.52 | 16.90 |
| *Phellinus igniarius （L. ex Fr.) Quel* (E3) | 500 | 96.38 | 19.28 |
| *Cucubalus baccifer L* (E4) | 500 | 95.36 | 19.07 |
| *Palhinhaea cernua (L.) Vasc. et Franco* (E5) | 500 | 104.25 | 20.85 |
| *Chloranthus henryi* (E6) | 500 | 97.14 | 19.428 |

**Table S2.** Components and their targets and scores are predicted by Swiss Target Prediction database.

| **Components** | **Targets** | **Probability*** |
| --- | --- | --- |
| Pernetic acid D | FNTA | 0.112041901 |
| Pernetic acid D | NOS2 | 0.112041901 |
| Pernetic acid D | TNF | 0.112041901 |
| Pernetic acid D | SRD5A2 | 0.112041901 |
| Pernetic acid D | PTGIR | 0.112041901 |
| Pernetic acid D | ITGAL | 0.112041901 |
| Pernetic acid D | NPC1L1 | 0.112041901 |
| Pernetic acid D | RORA | 0.112041901 |
| Pernetic acid D | HSD11B1 | 0.112041901 |
| Pernetic acid D | IDO1 | 0.112041901 |
| Pernetic acid D | PTGFR | 0.112041901 |
| Pernetic acid D | PTPN1 | 0.112041901 |
| Pernetic acid D | PTGER4 | 0.112041901 |
| Pernetic acid D | PTGER2 | 0.112041901 |
| Pernetic acid D | CYP19A1 | 0.112041901 |
| Pernetic acid D | CYP17A1 | 0.112041901 |
| Pernetic acid D | PTGS2 | 0.112041901 |
| Pernetic acid D | CPA1 | 0.112041901 |
| Pernetic acid D | NR3C1 | 0.112041901 |
| Pernetic acid D | FDFT1 | 0.112041901 |
| Pernetic acid D | SHBG | 0.112041901 |
| Pernetic acid D | PTGER3 | 0.112041901 |
| Pernetic acid D | LTB4R | 0.112041901 |
| Pernetic acid D | MME | 0.112041901 |
| Pernetic acid D | PINK1 | 0.112041901 |
| Pernetic acid D | CDK2 | 0.112041901 |
| Pernetic acid D | CTNNB1 | 0.112041901 |
| Pernetic acid D | PPARA | 0.112041901 |
| Pernetic acid D | PTGER1 | 0.112041901 |
| Pernetic acid D | AMPD3 | 0.112041901 |
| Pernetic acid D | CDC25A | 0.112041901 |
| Pernetic acid D | NR3C2 | 0.112041901 |
| Pernetic acid D | SERPINA6 | 0.112041901 |
| Pernetic acid D | NR1H3 | 0.112041901 |
| Pernetic acid D | SIGMAR1 | 0.112041901 |
| Pernetic acid D | LANCL2 | 0.112041901 |
| Pernetic acid D | PPARG | 0.112041901 |
| Pernetic acid D | PPARD | 0.112041901 |
| Pernetic acid D | SLC22A12 | 0.112041901 |
| Pernetic acid D | ITGAL ICAM1 ITGB2 | 0.112041901 |
| Pernetic acid D | TTR | 0.112041901 |
| Pernetic acid D | LTA4H | 0.112041901 |
| Pernetic acid D | HSPA1A | 0.112041901 |
| Pernetic acid D | PSEN2 | 0.112041901 |
| Pernetic acid D | AMPD2 | 0.112041901 |
| Pernetic acid D | HAO2 | 0.112041901 |
| Pernetic acid D | PIM1 | 0.112041901 |
| Pernetic acid D | PIM2 | 0.112041901 |
| Pernetic acid D | MAPK3 | 0.112041901 |
| Pernetic acid D | PRKCH | 0.112041901 |
| Pernetic acid D | HSD11B2 | 0.112041901 |
| Pernetic acid D | PRKCA | 0.112041901 |
| Gaultheroside A | TOP1 | 0.095623787 |
| Gaultheroside A | MMP13 | 0.095623787 |
| Gaultheroside A | MMP8 | 0.095623787 |
| Gaultherin | CA14 | 0.118883307 |
| Gaultherin | EPHX2 | 0.118883307 |
| Gaultherin | FUCA1 | 0.118883307 |
| Gaultherin | ADORA2A | 0.118883307 |
| Gaultherin | SLC29A1 | 0.118883307 |
| Gaultherin | ADORA3 | 0.118883307 |
| Gaultherin | TYR | 0.118883307 |
| Gaultherin | SLC5A2 | 0.118883307 |
| Gaultherin | AMY2A | 0.118883307 |
| Gaultherin | FOLH1 | 0.118883307 |
| Gaultherin | NEU2 | 0.118883307 |
| Gaultherin | NEU4 | 0.118883307 |
| Gaultherin | HPRT1 | 0.118883307 |
| Gaultherin B | CDK4 | 0.103186937 |
| Gaultherin B | BRAF | 0.103186937 |
| Gaultherin B | PDE5A | 0.103186937 |
| Gaultherin B | PITRM1 | 0.103186937 |
| Gaultherin B | PARP1 | 0.103186937 |
| Gaultherin B | DNM1 | 0.103186937 |
| Gaultherin B | TYMS | 0.103186937 |
| Gaultherin B | PDK1 | 0.103186937 |
| Gaultherin A | BRAF | 0.120225751 |
| Gaultherin A | ABCG2 | 0.120225751 |
| Gaultherin A | EPHB4 | 0.120225751 |
| Gaultherin A | CDK2 | 0.120225751 |
| Gaultherin A | CDK4 | 0.120225751 |
| Gaultherin A | PDK1 | 0.120225751 |
| Gaultherin A | CDK1 | 0.120225751 |
| Gaultherin A | CDK1 | 0.120225751 |
| Gaultherin A | CCNE2 | 0.120225751 |
| Gaultherin A | HSP90AA1 | 0.120225751 |
| Gaultherin A | PARP1 | 0.120225751 |
| Gaultherin A | SGK1 | 0.120225751 |
| Gaultherin A | EIF2AK2 | 0.120225751 |
| Gaultherin A | PLK1 | 0.120225751 |
| Gaultherin A | BMP1 | 0.120225751 |
| Gaultherin A | LYPLA1 | 0.120225751 |
| Gaultherin A | LYPLA2 | 0.120225751 |
| Gaultherin A | PIK3CD | 0.120225751 |
| Gaultherin A | MMP9 | 0.120225751 |
| Gaultherin A | MMP2 | 0.120225751 |
| Gaultherin A | PIK3CA | 0.120225751 |
| Gaultherin A | QPCTL | 0.120225751 |
| Gaultherin A | GCGR | 0.120225751 |
| Gaultherin A | STAT6 | 0.120225751 |
| Gaultherin A | PDE5A | 0.120225751 |
| Gaultherin A | MMP13 | 0.120225751 |
| Gaultherin A | MMP1 | 0.120225751 |
| Gaultherin A | BDKRB1 | 0.120225751 |
| Gaultherin A | DNM1 | 0.120225751 |
| Gaultherin A | AURKB | 0.120225751 |
| Gaultherin A | RPS6KB1 | 0.120225751 |
| Gaultherin A | AURKA | 0.120225751 |
| Gaultherin A | HSD17B1 | 0.120225751 |
| Gaultherin A | CCNE1 | 0.120225751 |
| Gaultherin A | CDK2 | 0.120225751 |
| Gaultherin A | TYMS | 0.120225751 |
| Gaultherin A | MMP3 | 0.120225751 |
| Gaultherin A | ASF1A | 0.120225751 |
| Gaultherin A | MKNK2 | 0.120225751 |
| Gaultherin A | PITRM1 | 0.120225751 |
| Gaultherin A | ADORA3 | 0.120225751 |
| Gaultherin A | MMP7 | 0.120225751 |
| Gaultherin A | MMP8 | 0.120225751 |
| Gaultherin A | MAP2K1 | 0.120225751 |
| Gaultherin A | MAPK3 | 0.120225751 |
| Gaultherin A | ABL1 | 0.120225751 |
| Gaultherin A | EPHA2 | 0.120225751 |
| Gaultherin A | TGM2 | 0.120225751 |
| Gaultherin A | EPHB2 | 0.120225751 |
| Gaultherin A | EPHA5 | 0.120225751 |
| Gaultherin A | EPHA4 | 0.120225751 |
| Gaultherin A | EPHA8 | 0.120225751 |
| Gaultherin A | EPHA7 | 0.120225751 |
| Gaultherin A | EPHB3 | 0.120225751 |
| Gaultherin A | EPHA3 | 0.120225751 |
| Gaultherin A | EPHB1 | 0.120225751 |
| Gaultherin A | EPHA1 | 0.120225751 |
| Gaultherin A | IMPDH1 | 0.120225751 |
| Gaultherin A | IMPDH2 | 0.120225751 |
| Gaultherin A | CA7 | 0.120225751 |
| Gaultherin A | CA12 | 0.120225751 |
| Gaultherin A | CA14 | 0.120225751 |
| Gaultherin A | CA5B | 0.120225751 |
| Gaultherin A | CA5A | 0.120225751 |
| Gaultheric acid | POLA1 | 0.618201678 |
| Gaultheric acid | HSD17B1 | 0.134939009 |
| Gaultheric acid | TBXA2R | 0.118277085 |
| Gaultheric acid | ESR1 | 0.101613855 |
| Gaultheric acid | ESR2 | 0.101613855 |
| Gaultheric acid | STS | 0.101613855 |
| Gaultheric acid | PTGDR | 0.101613855 |
| Gaultheric acid | NR1H3 | 0.101613855 |
| Gaultheric acid | NR1H2 | 0.101613855 |
| Gaultheric acid | NR3C1 | 0.101613855 |
| Gaultheric acid | CYP19A1 | 0.101613855 |
| Gaultheric acid | AMPD2 | 0.101613855 |
| Gaultheric acid | TP53 | 0.101613855 |
| Gaultheric acid | AGTR1 | 0.101613855 |
| Gaultheric acid | RXRA | 0.101613855 |
| Gaultheric acid | MME | 0.101613855 |
| Gaultheric acid | ECE1 | 0.101613855 |
| Gaultheric acid | CTSA | 0.101613855 |
| Gaultheric acid | SHBG | 0.101613855 |
| Gaultheric acid | AKR1C3 | 0.101613855 |
| Gaultheric acid | KDR | 0.101613855 |
| Gaultheric acid | SLC6A4 | 0.101613855 |
| Gaultheric acid | P2RX3 | 0.101613855 |
| Gaultheric acid | ESRRA | 0.101613855 |
| Gaultheric acid | ESRRB | 0.101613855 |
| Gaultheric acid | GPER1 | 0.101613855 |
| Gaultheric acid | HTR2B | 0.101613855 |
| Gaultheric acid | ITGB1 ITGA4 | 0.101613855 |
| Gaultheric acid | FLT1 | 0.101613855 |
| Gaultheric acid | BACE1 | 0.101613855 |
| Gaultheric acid | MDM2 | 0.101613855 |
| Gaultheric acid | AMPD1 | 0.101613855 |
| Gaultheric acid | AMPD3 | 0.101613855 |
| Gaultheric acid | ADCY10 | 0.101613855 |
| Gaultheric acid | CYP24A1 | 0.101613855 |
| Gaultheric acid | GSK3B | 0.101613855 |
| Gaultheric acid | GSK3A | 0.101613855 |
| Gaultheric acid | HCAR2 | 0.101613855 |
| Gaultheric acid | MKNK2 | 0.101613855 |
| Gaultheric acid | ACE2 | 0.101613855 |
| Gaultheric acid | ADAMTS5 | 0.101613855 |
| Gaultheric acid | MMP14 | 0.101613855 |
| Gaultheric acid | AURKA | 0.101613855 |
| Gaultheric acid | PDE5A | 0.101613855 |
| Gaultheric acid | PTGES | 0.101613855 |
| Gaultheric acid | PSEN2 | 0.101613855 |
| Gaultheric acid | BCL2L2 | 0.101613855 |
| Gaultheric acid | PTPRG | 0.101613855 |
| Dhasingreoside | NMUR2 | 0.227065841 |
| Dhasingreoside | ADRA2A | 0.227065841 |
| Dhasingreoside | ADRA2C | 0.227065841 |
| Dhasingreoside | ACHE | 0.227065841 |
| Dhasingreoside | RPS6KA3 | 0.179283317 |
| Dhasingreoside | PTGS2 | 0.160113589 |
| Dhasingreoside | NQO2 | 0.103119912 |
| Dhasingreoside | AKR1B1 | 0.074717926 |
| Dhasingreoside | NOX4 | 0.074717926 |
| Dhasingreoside | CA2 | 0.065155937 |
| Dhasingreoside | CA7 | 0.065155937 |
| Dhasingreoside | CA12 | 0.065155937 |
| Dhasingreoside | CA4 | 0.065155937 |
| Dhasingreoside | XDH | 0.065155937 |
| Dhasingreoside | CD38 | 0.05564795 |
| Dhasingreoside | PDE5A | 0.05564795 |
| Dhasingreoside | TNF | 0.05564795 |
| Dhasingreoside | IL2 | 0.05564795 |
| Dhasingreoside | ADORA1 | 0.05564795 |
| Dhasingreoside | ALOX5 | 0.05564795 |
| Arbutin | AKR1B1 | 0.112041901 |
| Arbutin | TYR | 0.112041901 |
| Arbutin | ADORA2A | 0.112041901 |
| Arbutin | ADA | 0.112041901 |
| Arbutin | PNP | 0.112041901 |
| Arbutin | CA6 | 0.112041901 |
| Arbutin | CA4 | 0.112041901 |
| Arbutin | CA5A | 0.112041901 |
| Arbutin | CA14 | 0.112041901 |
| Arbutin | P2RX3 | 0.112041901 |
| Arbutin | CDA | 0.112041901 |
| Arbutin | SLC5A2 | 0.112041901 |
| Arbutin | ADORA3 | 0.112041901 |
| Arbutin | ADK | 0.112041901 |
| Arbutin | DPP4 | 0.112041901 |
| 9-O-Benzoyl-lariciresinol | MCL1 | 0.120225751 |
| 9-O-Benzoyl-lariciresinol | PTAFR | 0.120225751 |
| 9-O-Benzoyl-lariciresinol | ALOX5 | 0.120225751 |
| 9-O-Benzoyl-lariciresinol | MAPK9 | 0.120225751 |
| 9-O-Benzoyl-lariciresinol | ROCK2 | 0.120225751 |
| 9-O-Benzoyl-lariciresinol | TDP1 | 0.120225751 |
| 9-O-Benzoyl-lariciresinol | SHBG | 0.120225751 |
| 9-O-Benzoyl-lariciresinol | KCNA5 | 0.120225751 |
| 9-O-Benzoyl-lariciresinol | PDK1 | 0.120225751 |
| 9-O-Benzoyl-lariciresinol | ROCK1 | 0.120225751 |
| 9-O-Benzoyl-lariciresinol | HDAC8 | 0.120225751 |
| 9-O-Benzoyl-lariciresinol | PDE5A | 0.120225751 |
| 9-O-Benzoyl-lariciresinol | CASP3 | 0.120225751 |
| 9-O-Benzoyl-lariciresinol | CTSS | 0.120225751 |
| 9-O-Benzoyl-lariciresinol | CASP6 | 0.120225751 |
| 9-O-Benzoyl-lariciresinol | CASP7 | 0.120225751 |
| 9-O-Benzoyl-lariciresinol | CASP8 | 0.120225751 |
| 9-O-Benzoyl-lariciresinol | CTSL | 0.120225751 |
| 9-O-Benzoyl-lariciresinol | CTSB | 0.120225751 |
| 9-O-Benzoyl-lariciresinol | CASP1 | 0.120225751 |
| 9-O-Benzoyl-lariciresinol | SERPINE1 | 0.120225751 |
| 9-O-Benzoyl-lariciresinol | P2RX3 | 0.120225751 |
| 9-O-Benzoyl-lariciresinol | PIK3CA | 0.120225751 |
| 9-O-Benzoyl-lariciresinol | ABCB1 | 0.120225751 |
| 9-O-Benzoyl-lariciresinol | ALOX5AP | 0.120225751 |
| 9-O-Benzoyl-lariciresinol | HDAC3 | 0.120225751 |
| 9-O-Benzoyl-lariciresinol | HDAC6 | 0.120225751 |
| 9-O-Benzoyl-lariciresinol | HDAC1 | 0.120225751 |
| 9-O-Benzoyl-lariciresinol | BMP1 | 0.120225751 |
| 9-O-Benzoyl-lariciresinol | JAK3 | 0.120225751 |
| 9-O-Benzoyl-lariciresinol | JAK1 | 0.120225751 |
| 9-O-Benzoyl-lariciresinol | JAK2 | 0.120225751 |
| 9-O-Benzoyl-lariciresinol | MMP8 | 0.120225751 |
| 9-O-Benzoyl-lariciresinol | HDAC2 | 0.120225751 |
| 9-O-Benzoyl-lariciresinol | ADAM10 | 0.120225751 |
| 5-Hydroxy-4',7-dimethoxy-6-methylflavone | ABCG2 | 0.384850756 |
| 5-Hydroxy-4',7-dimethoxy-6-methylflavone | ABCB1 | 0.184930052 |
| 5-Hydroxy-4',7-dimethoxy-6-methylflavone | AKR1B1 | 0.168264714 |
| 5-Hydroxy-4',7-dimethoxy-6-methylflavone | SYK | 0.143269257 |
| 5-Hydroxy-4',7-dimethoxy-6-methylflavone | MAOA | 0.134939009 |
| 5-Hydroxy-4',7-dimethoxy-6-methylflavone | ADORA1 | 0.134939009 |
| 5-Hydroxy-4',7-dimethoxy-6-methylflavone | ADORA2A | 0.134939009 |
| 5-Hydroxy-4',7-dimethoxy-6-methylflavone | ACHE | 0.126610169 |
| 5-Hydroxy-4',7-dimethoxy-6-methylflavone | OPRD1 | 0.126610169 |
| 5-Hydroxy-4',7-dimethoxy-6-methylflavone | HSD17B1 | 0.118277085 |
| 5-Hydroxy-4',7-dimethoxy-6-methylflavone | ADORA3 | 0.118277085 |
| 5-Hydroxy-4',7-dimethoxy-6-methylflavone | ALOX15 | 0.118277085 |
| 5-Hydroxy-4',7-dimethoxy-6-methylflavone | APP | 0.118277085 |
| 5-Hydroxy-4',7-dimethoxy-6-methylflavone | ALOX5 | 0.118277085 |
| 5-Hydroxy-4',7-dimethoxy-6-methylflavone | ESR2 | 0.118277085 |
| 5-Hydroxy-4',7-dimethoxy-6-methylflavone | LCK | 0.118277085 |
| 5-Hydroxy-4',7-dimethoxy-6-methylflavone | PIM1 | 0.10994577 |
| 5-Hydroxy-4',7-dimethoxy-6-methylflavone | CDK1 | 0.10994577 |
| 5-Hydroxy-4',7-dimethoxy-6-methylflavone | PTGS2 | 0.10994577 |
| 5-Hydroxy-4',7-dimethoxy-6-methylflavone | CYP1B1 | 0.10994577 |
| 5-Hydroxy-4',7-dimethoxy-6-methylflavone | MMP9 | 0.10994577 |
| 5-Hydroxy-4',7-dimethoxy-6-methylflavone | NOX4 | 0.10994577 |
| 5-Hydroxy-4',7-dimethoxy-6-methylflavone | NAE1 | 0.10994577 |
| 5-Hydroxy-4',7-dimethoxy-6-methylflavone | KIT | 0.10994577 |
| 5-Hydroxy-4',7-dimethoxy-6-methylflavone | AKR1B10 | 0.10994577 |
| 5-Hydroxy-4',7-dimethoxy-6-methylflavone | CA2 | 0.10994577 |
| 5-Hydroxy-4',7-dimethoxy-6-methylflavone | CA1 | 0.10994577 |
| 5-Hydroxy-4',7-dimethoxy-6-methylflavone | ESR1 | 0.10994577 |
| 5-Hydroxy-4',7-dimethoxy-6-methylflavone | PTPRS | 0.10994577 |
| 5-Hydroxy-4',7-dimethoxy-6-methylflavone | AMY1A | 0.10994577 |
| 5-Hydroxy-4',7-dimethoxy-6-methylflavone | GSK3B | 0.10994577 |
| 5-Hydroxy-4',7-dimethoxy-6-methylflavone | CA7 | 0.10994577 |
| 5-Hydroxy-4',7-dimethoxy-6-methylflavone | CA12 | 0.10994577 |
| 5-Hydroxy-4',7-dimethoxy-6-methylflavone | CA9 | 0.10994577 |
| 5-Hydroxy-4',7-dimethoxy-6-methylflavone | CBR1 | 0.10994577 |
| 5-Hydroxy-4',7-dimethoxy-6-methylflavone | AR | 0.10994577 |
| 5-Hydroxy-4',7-dimethoxy-6-methylflavone | PLG | 0.10994577 |
| 5-Hydroxy-4',7-dimethoxy-6-methylflavone | PARP1 | 0.10994577 |
| 5-Hydroxy-4',7-dimethoxy-6-methylflavone | CCNB3 | 0.10994577 |
| 5-Hydroxy-4',7-dimethoxy-6-methylflavone | MMP2 | 0.101613855 |
| 5-Hydroxy-4',7-dimethoxy-6-methylflavone | MAOB | 0.101613855 |
| 5-Hydroxy-4',7-dimethoxy-6-methylflavone | CDK5R1 | 0.101613855 |
| 5-Hydroxy-4',7-dimethoxy-6-methylflavone | ABCC1 | 0.101613855 |
| 5-Hydroxy-4',7-dimethoxy-6-methylflavone | MCL1 | 0.101613855 |
| 5-Hydroxy-4',7-dimethoxy-6-methylflavone | CSNK2A1 | 0.101613855 |
| 5-Hydroxy-4',7-dimethoxy-6-methylflavone | GLO1 | 0.101613855 |
| 5-Hydroxy-4',7-dimethoxy-6-methylflavone | MMP12 | 0.101613855 |
| 5-Hydroxy-4',7-dimethoxy-6-methylflavone | ARG1 | 0.101613855 |
| 5-Hydroxy-4',7-dimethoxy-6-methylflavone | HSD17B2 | 0.101613855 |
| 5-Hydroxy-4',7-dimethoxy-6-methylflavone | KDM4E | 0.101613855 |
| 5-Hydroxy-4',7-dimethoxy-6-methylflavone | FLT3 | 0.101613855 |
| 5-Hydroxy-4',7-dimethoxy-6-methylflavone | BACE1 | 0.101613855 |
| 5-Hydroxy-4',7-dimethoxy-6-methylflavone | CYP19A1 | 0.101613855 |
| 5-Hydroxy-4',7-dimethoxy-6-methylflavone | PTPN1 | 0.101613855 |
| 5-Hydroxy-4',7-dimethoxy-6-methylflavone | BCHE | 0.101613855 |
| 5-Hydroxy-4',7-dimethoxy-6-methylflavone | TNKS2 | 0.101613855 |
| 5-Hydroxy-4',7-dimethoxy-6-methylflavone | TNKS | 0.101613855 |
| 5-Hydroxy-4',7-dimethoxy-6-methylflavone | AHR | 0.101613855 |
| 5-Hydroxy-4',7-dimethoxy-6-methylflavone | XDH | 0.101613855 |
| 5-Hydroxy-4',7-dimethoxy-6-methylflavone | OPRM1 | 0.101613855 |
| 5-Hydroxy-4',7-dimethoxy-6-methylflavone | MMP3 | 0.101613855 |
| 5-Hydroxy-4',7-dimethoxy-6-methylflavone | CA4 | 0.101613855 |
| 5-Hydroxy-4',7-dimethoxy-6-methylflavone | CFTR | 0.101613855 |
| 5-Hydroxy-4',7-dimethoxy-6-methylflavone | CDK2 | 0.101613855 |
| 5-Hydroxy-4',7-dimethoxy-6-methylflavone | PFKFB3 | 0.101613855 |
| 5-Hydroxy-4',7-dimethoxy-6-methylflavone | TTR | 0.101613855 |
| 5-Hydroxy-4',7-dimethoxy-6-methylflavone | SIGMAR1 | 0.101613855 |
| 5-Hydroxy-4',7-dimethoxy-6-methylflavone | MMP13 | 0.101613855 |
| 5-Hydroxy-4',7-dimethoxy-6-methylflavone | MET | 0.101613855 |
| 5-Hydroxy-4',7-dimethoxy-6-methylflavone | MPG | 0.101613855 |
| 5-Hydroxy-4',7-dimethoxy-6-methylflavone | SLC22A12 | 0.101613855 |
| 5-Hydroxy-4',7-dimethoxy-6-methylflavone | AURKB | 0.101613855 |
| 5-Hydroxy-4',7-dimethoxy-6-methylflavone | TERT | 0.101613855 |
| 5-Hydroxy-4',7-dimethoxy-6-methylflavone | CDK6 | 0.101613855 |
| 5-Hydroxy-4',7-dimethoxy-6-methylflavone | CA6 | 0.101613855 |
| 5-Hydroxy-4',7-dimethoxy-6-methylflavone | CA14 | 0.101613855 |
| 5-Hydroxy-4',7-dimethoxy-6-methylflavone | CA13 | 0.101613855 |
| 5-Hydroxy-4',7-dimethoxy-6-methylflavone | CA5A | 0.101613855 |
| 5-Hydroxy-4',7-dimethoxy-6-methylflavone | MPO | 0.101613855 |
| 5-Hydroxy-4',7-dimethoxy-6-methylflavone | CA3 | 0.101613855 |
| 5-Hydroxy-4',7-dimethoxy-6-methylflavone | PKN1 | 0.101613855 |
| 5-Hydroxy-4',7-dimethoxy-6-methylflavone | NEK6 | 0.101613855 |
| 5-Hydroxy-4',7-dimethoxy-6-methylflavone | APEX1 | 0.101613855 |
| 5-Hydroxy-4',7-dimethoxy-6-methylflavone | AKR1C2 | 0.101613855 |
| 5-Hydroxy-4',7-dimethoxy-6-methylflavone | AKR1C1 | 0.101613855 |
| 5-Hydroxy-4',7-dimethoxy-6-methylflavone | AKR1C4 | 0.101613855 |
| 5-Hydroxy-4',7-dimethoxy-6-methylflavone | AKR1A1 | 0.101613855 |
| 5-Hydroxy-4',7-dimethoxy-6-methylflavone | SRC | 0.101613855 |
| 5-Hydroxy-4',7-dimethoxy-6-methylflavone | PLK1 | 0.101613855 |
| 5-Hydroxy-4',7-dimethoxy-6-methylflavone | EGFR | 0.101613855 |
| 5-Hydroxy-4',7-dimethoxy-6-methylflavone | KDR | 0.101613855 |
| 5-Hydroxy-4',7-dimethoxy-6-methylflavone | ST6GAL1 | 0.101613855 |
| 5-Hydroxy-4',7-dimethoxy-6-methylflavone | NOS2 | 0.101613855 |
| 5-Hydroxy-4',7-dimethoxy-6-methylflavone | PIK3CG | 0.101613855 |
| 5-Hydroxy-4',7-dimethoxy-6-methylflavone | F2 | 0.101613855 |
| 5-Hydroxy-4',7-dimethoxy-6-methylflavone | KDM5A | 0.101613855 |
| 5-Hydroxy-4',7-dimethoxy-6-methylflavone | IGF1R | 0.101613855 |
| 5-Hydroxy-4',7-dimethoxy-6-methylflavone | PTK2 | 0.101613855 |
| 5-Hydroxy-4',7-dimethoxy-6-methylflavone | FYN | 0.101613855 |
| 5-Hydroxy-4',7-dimethoxy-6-methylflavone | GRK6 | 0.101613855 |
| 4',5-Dihydroxy-3,7-dimethoxy-6-methylflavone(8-Demethyllatifolin) | AKR1B1 | 0.233887255 |
| 4',5-Dihydroxy-3,7-dimethoxy-6-methylflavone(8-Demethyllatifolin) | ADORA3 | 0.217731049 |
| 4',5-Dihydroxy-3,7-dimethoxy-6-methylflavone(8-Demethyllatifolin) | ABCB1 | 0.201584698 |
| 4',5-Dihydroxy-3,7-dimethoxy-6-methylflavone(8-Demethyllatifolin) | NOX4 | 0.177362767 |
| 4',5-Dihydroxy-3,7-dimethoxy-6-methylflavone(8-Demethyllatifolin) | ABCG2 | 0.177362767 |
| 4',5-Dihydroxy-3,7-dimethoxy-6-methylflavone(8-Demethyllatifolin) | ADORA1 | 0.161187413 |
| 4',5-Dihydroxy-3,7-dimethoxy-6-methylflavone(8-Demethyllatifolin) | BACE1 | 0.153092564 |
| 4',5-Dihydroxy-3,7-dimethoxy-6-methylflavone(8-Demethyllatifolin) | ADORA2A | 0.136969565 |
| 4',5-Dihydroxy-3,7-dimethoxy-6-methylflavone(8-Demethyllatifolin) | MCL1 | 0.120823672 |
| 4',5-Dihydroxy-3,7-dimethoxy-6-methylflavone(8-Demethyllatifolin) | ALK | 0.120823672 |
| 4',5-Dihydroxy-3,7-dimethoxy-6-methylflavone(8-Demethyllatifolin) | AKT1 | 0.112748418 |
| 4',5-Dihydroxy-3,7-dimethoxy-6-methylflavone(8-Demethyllatifolin) | OPRD1 | 0.112748418 |
| 4',5-Dihydroxy-3,7-dimethoxy-6-methylflavone(8-Demethyllatifolin) | ESR2 | 0.112748418 |
| 4',5-Dihydroxy-3,7-dimethoxy-6-methylflavone(8-Demethyllatifolin) | TYR | 0.112748418 |
| 4',5-Dihydroxy-3,7-dimethoxy-6-methylflavone(8-Demethyllatifolin) | CA7 | 0.112748418 |
| 4',5-Dihydroxy-3,7-dimethoxy-6-methylflavone(8-Demethyllatifolin) | ABCC1 | 0.112748418 |
| 4',5-Dihydroxy-3,7-dimethoxy-6-methylflavone(8-Demethyllatifolin) | AHR | 0.112748418 |
| 4',5-Dihydroxy-3,7-dimethoxy-6-methylflavone(8-Demethyllatifolin) | ESRRA | 0.112748418 |
| 4',5-Dihydroxy-3,7-dimethoxy-6-methylflavone(8-Demethyllatifolin) | BCHE | 0.104671941 |
| 4',5-Dihydroxy-3,7-dimethoxy-6-methylflavone(8-Demethyllatifolin) | ACHE | 0.104671941 |
| 4',5-Dihydroxy-3,7-dimethoxy-6-methylflavone(8-Demethyllatifolin) | HSD17B2 | 0.104671941 |
| 4',5-Dihydroxy-3,7-dimethoxy-6-methylflavone(8-Demethyllatifolin) | HSD17B1 | 0.104671941 |
| 4',5-Dihydroxy-3,7-dimethoxy-6-methylflavone(8-Demethyllatifolin) | PLG | 0.104671941 |
| 4',5-Dihydroxy-3,7-dimethoxy-6-methylflavone(8-Demethyllatifolin) | PTPRS | 0.104671941 |
| 4',5-Dihydroxy-3,7-dimethoxy-6-methylflavone(8-Demethyllatifolin) | DAPK1 | 0.104671941 |
| 4',5-Dihydroxy-3,7-dimethoxy-6-methylflavone(8-Demethyllatifolin) | MPG | 0.104671941 |
| 4',5-Dihydroxy-3,7-dimethoxy-6-methylflavone(8-Demethyllatifolin) | GSK3B | 0.104671941 |
| 4',5-Dihydroxy-3,7-dimethoxy-6-methylflavone(8-Demethyllatifolin) | SLC22A12 | 0.104671941 |
| 4',5-Dihydroxy-3,7-dimethoxy-6-methylflavone(8-Demethyllatifolin) | KIT | 0.104671941 |
| 4',5-Dihydroxy-3,7-dimethoxy-6-methylflavone(8-Demethyllatifolin) | CYP1B1 | 0.104671941 |
| 4',5-Dihydroxy-3,7-dimethoxy-6-methylflavone(8-Demethyllatifolin) | MAPT | 0.104671941 |
| 4',5-Dihydroxy-3,7-dimethoxy-6-methylflavone(8-Demethyllatifolin) | KDM4E | 0.104671941 |
| 4',5-Dihydroxy-3,7-dimethoxy-6-methylflavone(8-Demethyllatifolin) | TOP2A | 0.104671941 |
| 4',5-Dihydroxy-3,7-dimethoxy-6-methylflavone(8-Demethyllatifolin) | MYLK | 0.104671941 |
| 4',5-Dihydroxy-3,7-dimethoxy-6-methylflavone(8-Demethyllatifolin) | MPO | 0.104671941 |
| 4',5-Dihydroxy-3,7-dimethoxy-6-methylflavone(8-Demethyllatifolin) | PIK3R1 | 0.104671941 |
| 4',5-Dihydroxy-3,7-dimethoxy-6-methylflavone(8-Demethyllatifolin) | PYGL | 0.104671941 |
| 4',5-Dihydroxy-3,7-dimethoxy-6-methylflavone(8-Demethyllatifolin) | SYK | 0.104671941 |
| 4',5-Dihydroxy-3,7-dimethoxy-6-methylflavone(8-Demethyllatifolin) | MMP13 | 0.104671941 |
| 4',5-Dihydroxy-3,7-dimethoxy-6-methylflavone(8-Demethyllatifolin) | MMP3 | 0.104671941 |
| 4',5-Dihydroxy-3,7-dimethoxy-6-methylflavone(8-Demethyllatifolin) | CA3 | 0.104671941 |
| 4',5-Dihydroxy-3,7-dimethoxy-6-methylflavone(8-Demethyllatifolin) | CA14 | 0.104671941 |
| 4',5-Dihydroxy-3,7-dimethoxy-6-methylflavone(8-Demethyllatifolin) | CSNK2A1 | 0.104671941 |
| 4',5-Dihydroxy-3,7-dimethoxy-6-methylflavone(8-Demethyllatifolin) | CA13 | 0.104671941 |
| 4',5-Dihydroxy-3,7-dimethoxy-6-methylflavone(8-Demethyllatifolin) | PLA2G1B | 0.104671941 |
| 4',5-Dihydroxy-3,7-dimethoxy-6-methylflavone(8-Demethyllatifolin) | CA5A | 0.104671941 |
| 4',5-Dihydroxy-3,7-dimethoxy-6-methylflavone(8-Demethyllatifolin) | APEX1 | 0.104671941 |
| 4',5-Dihydroxy-3,7-dimethoxy-6-methylflavone(8-Demethyllatifolin) | AKR1C2 | 0.104671941 |
| 4',5-Dihydroxy-3,7-dimethoxy-6-methylflavone(8-Demethyllatifolin) | AKR1C1 | 0.104671941 |
| 4',5-Dihydroxy-3,7-dimethoxy-6-methylflavone(8-Demethyllatifolin) | AKR1C3 | 0.104671941 |
| 4',5-Dihydroxy-3,7-dimethoxy-6-methylflavone(8-Demethyllatifolin) | AKR1C4 | 0.104671941 |
| 4',5-Dihydroxy-3,7-dimethoxy-6-methylflavone(8-Demethyllatifolin) | AKR1A1 | 0.104671941 |
| 4',5-Dihydroxy-3,7-dimethoxy-6-methylflavone(8-Demethyllatifolin) | CA2 | 0.104671941 |
| 4',5-Dihydroxy-3,7-dimethoxy-6-methylflavone(8-Demethyllatifolin) | ALOX15 | 0.104671941 |
| 4',5-Dihydroxy-3,7-dimethoxy-6-methylflavone(8-Demethyllatifolin) | ALOX12 | 0.104671941 |
| 4',5-Dihydroxy-3,7-dimethoxy-6-methylflavone(8-Demethyllatifolin) | MMP9 | 0.104671941 |
| 4',5-Dihydroxy-3,7-dimethoxy-6-methylflavone(8-Demethyllatifolin) | MMP2 | 0.104671941 |
| 4',5-Dihydroxy-3,7-dimethoxy-6-methylflavone(8-Demethyllatifolin) | NAE1 | 0.104671941 |
| 4',5-Dihydroxy-3,7-dimethoxy-6-methylflavone(8-Demethyllatifolin) | XDH | 0.104671941 |
| 4',5-Dihydroxy-3,7-dimethoxy-6-methylflavone(8-Demethyllatifolin) | ESR1 | 0.104671941 |
| 4',5-Dihydroxy-3,7-dimethoxy-6-methylflavone(8-Demethyllatifolin) | FLT3 | 0.104671941 |
| 4',5-Dihydroxy-3,7-dimethoxy-6-methylflavone(8-Demethyllatifolin) | CDK1 | 0.104671941 |
| 4',5-Dihydroxy-3,7-dimethoxy-6-methylflavone(8-Demethyllatifolin) | ODC1 | 0.104671941 |
| 4',5-Dihydroxy-3,7-dimethoxy-6-methylflavone(8-Demethyllatifolin) | PFKFB3 | 0.104671941 |
| 4',5-Dihydroxy-3,7-dimethoxy-6-methylflavone(8-Demethyllatifolin) | CA4 | 0.104671941 |
| 4',5-Dihydroxy-3,7-dimethoxy-6-methylflavone(8-Demethyllatifolin) | TNKS2 | 0.104671941 |
| 4',5-Dihydroxy-3,7-dimethoxy-6-methylflavone(8-Demethyllatifolin) | TNKS | 0.104671941 |
| 4',5-Dihydroxy-3,7-dimethoxy-6-methylflavone(8-Demethyllatifolin) | APP | 0.104671941 |
| 4',5-Dihydroxy-3,7-dimethoxy-6-methylflavone(8-Demethyllatifolin) | ARG1 | 0.104671941 |
| 4',5-Dihydroxy-3,7-dimethoxy-6-methylflavone(8-Demethyllatifolin) | PDE5A | 0.104671941 |
| 4',5-Dihydroxy-3,7-dimethoxy-6-methylflavone(8-Demethyllatifolin) | PIM1 | 0.104671941 |
| 4',5-Dihydroxy-3,7-dimethoxy-6-methylflavone(8-Demethyllatifolin) | NOS2 | 0.104671941 |
| 4',5-Dihydroxy-3,7-dimethoxy-6-methylflavone(8-Demethyllatifolin) | CA12 | 0.104671941 |
| 4',5-Dihydroxy-3,7-dimethoxy-6-methylflavone(8-Demethyllatifolin) | OPRM1 | 0.104671941 |
| 4',5-Dihydroxy-3,7-dimethoxy-6-methylflavone(8-Demethyllatifolin) | TTR | 0.104671941 |
| 4',5-Dihydroxy-3,7-dimethoxy-6-methylflavone(8-Demethyllatifolin) | AKR1B10 | 0.104671941 |
| 4',5-Dihydroxy-3,7-dimethoxy-6-methylflavone(8-Demethyllatifolin) | MAOA | 0.104671941 |
| 4',5-Dihydroxy-3,7-dimethoxy-6-methylflavone(8-Demethyllatifolin) | CA6 | 0.104671941 |
| 4',5-Dihydroxy-3,7-dimethoxy-6-methylflavone(8-Demethyllatifolin) | AMY1A | 0.104671941 |
| 4',5-Dihydroxy-3,7-dimethoxy-6-methylflavone(8-Demethyllatifolin) | GRK6 | 0.104671941 |
| 4',5-Dihydroxy-3,7-dimethoxy-6-methylflavone(8-Demethyllatifolin) | EGFR | 0.104671941 |
| 4',5-Dihydroxy-3,7-dimethoxy-6-methylflavone(8-Demethyllatifolin) | ALOX5 | 0.104671941 |
| 4',5-Dihydroxy-3,7-dimethoxy-6-methylflavone(8-Demethyllatifolin) | AVPR2 | 0.104671941 |
| 4',5-Dihydroxy-3,7-dimethoxy-6-methylflavone(8-Demethyllatifolin) | PIK3CG | 0.104671941 |
| 4',5-Dihydroxy-3,7-dimethoxy-6-methylflavone(8-Demethyllatifolin) | CXCR1 | 0.104671941 |
| 4',5-Dihydroxy-3,7-dimethoxy-6-methylflavone(8-Demethyllatifolin) | PTGS2 | 0.104671941 |
| 4',5-Dihydroxy-3,7-dimethoxy-6-methylflavone(8-Demethyllatifolin) | PTPN1 | 0.104671941 |
| 4',5-Dihydroxy-3,7-dimethoxy-6-methylflavone(8-Demethyllatifolin) | CYP19A1 | 0.104671941 |
| 4',5-Dihydroxy-3,7-dimethoxy-6-methylflavone(8-Demethyllatifolin) | F2 | 0.104671941 |
| 4',5-Dihydroxy-3,7-dimethoxy-6-methylflavone(8-Demethyllatifolin) | AR | 0.104671941 |
| 4',5-Dihydroxy-3,7-dimethoxy-6-methylflavone(8-Demethyllatifolin) | CFTR | 0.104671941 |
| 4',5-Dihydroxy-3,7-dimethoxy-6-methylflavone(8-Demethyllatifolin) | IGF1R | 0.104671941 |
| 4',5-Dihydroxy-3,7-dimethoxy-6-methylflavone(8-Demethyllatifolin) | AURKB | 0.104671941 |
| 4',5-Dihydroxy-3,7-dimethoxy-6-methylflavone(8-Demethyllatifolin) | SRC | 0.104671941 |
| 4',5-Dihydroxy-3,7-dimethoxy-6-methylflavone(8-Demethyllatifolin) | PTK2 | 0.104671941 |
| 4',5-Dihydroxy-3,7-dimethoxy-6-methylflavone(8-Demethyllatifolin) | KDR | 0.104671941 |
| 4',5-Dihydroxy-3,7-dimethoxy-6-methylflavone(8-Demethyllatifolin) | PLK1 | 0.104671941 |
| 4',5-Dihydroxy-3,7-dimethoxy-6-methylflavone(8-Demethyllatifolin) | PKN1 | 0.104671941 |
| 4',5-Dihydroxy-3,7-dimethoxy-6-methylflavone(8-Demethyllatifolin) | MET | 0.104671941 |
| 4',5-Dihydroxy-3,7-dimethoxy-6-methylflavone(8-Demethyllatifolin) | NEK2 | 0.104671941 |
| 4',5-Dihydroxy-3,7-dimethoxy-6-methylflavone(8-Demethyllatifolin) | ABCG2 | 0.514392373 |
| 4',5-Dihydroxy-3,7-dimethoxy-6-methylflavone(8-Demethyllatifolin) | AKR1B1 | 0.237888614 |
| 4',5-Dihydroxy-3,7-dimethoxy-6-methylflavone(8-Demethyllatifolin) | ADORA1 | 0.214178827 |
| 4',5-Dihydroxy-3,7-dimethoxy-6-methylflavone(8-Demethyllatifolin) | ADORA2A | 0.214178827 |
| 4',5-Dihydroxy-3,7-dimethoxy-6-methylflavone(8-Demethyllatifolin) | ADORA3 | 0.214178827 |
| 4',5-Dihydroxy-3,7-dimethoxy-6-methylflavone(8-Demethyllatifolin) | HSD17B1 | 0.214178827 |
| 4',5-Dihydroxy-3,7-dimethoxy-6-methylflavone(8-Demethyllatifolin) | SYK | 0.166798772 |
| 4',5-Dihydroxy-3,7-dimethoxy-6-methylflavone(8-Demethyllatifolin) | OPRD1 | 0.158886034 |
| 4',5-Dihydroxy-3,7-dimethoxy-6-methylflavone(8-Demethyllatifolin) | CYP1B1 | 0.15098181 |
| 4',5-Dihydroxy-3,7-dimethoxy-6-methylflavone(8-Demethyllatifolin) | ESR2 | 0.15098181 |
| 4',5-Dihydroxy-3,7-dimethoxy-6-methylflavone(8-Demethyllatifolin) | ABCB1 | 0.143102156 |
| 4',5-Dihydroxy-3,7-dimethoxy-6-methylflavone(8-Demethyllatifolin) | FLT3 | 0.143102156 |
| 4',5-Dihydroxy-3,7-dimethoxy-6-methylflavone(8-Demethyllatifolin) | CSNK2A1 | 0.143102156 |
| 4',5-Dihydroxy-3,7-dimethoxy-6-methylflavone(8-Demethyllatifolin) | PIM1 | 0.143102156 |
| 4',5-Dihydroxy-3,7-dimethoxy-6-methylflavone(8-Demethyllatifolin) | ESR1 | 0.135202128 |
| 4',5-Dihydroxy-3,7-dimethoxy-6-methylflavone(8-Demethyllatifolin) | PLG | 0.135202128 |
| 4',5-Dihydroxy-3,7-dimethoxy-6-methylflavone(8-Demethyllatifolin) | KIT | 0.135202128 |
| 4',5-Dihydroxy-3,7-dimethoxy-6-methylflavone(8-Demethyllatifolin) | CYP19A1 | 0.135202128 |
| 4',5-Dihydroxy-3,7-dimethoxy-6-methylflavone(8-Demethyllatifolin) | AKR1B10 | 0.135202128 |
| 4',5-Dihydroxy-3,7-dimethoxy-6-methylflavone(8-Demethyllatifolin) | CDK6 | 0.12730257 |
| 4',5-Dihydroxy-3,7-dimethoxy-6-methylflavone(8-Demethyllatifolin) | CFTR | 0.12730257 |
| 4',5-Dihydroxy-3,7-dimethoxy-6-methylflavone(8-Demethyllatifolin) | MAOA | 0.12730257 |
| 4',5-Dihydroxy-3,7-dimethoxy-6-methylflavone(8-Demethyllatifolin) | GSK3B | 0.12730257 |
| 4',5-Dihydroxy-3,7-dimethoxy-6-methylflavone(8-Demethyllatifolin) | HSD17B2 | 0.12730257 |
| 4',5-Dihydroxy-3,7-dimethoxy-6-methylflavone(8-Demethyllatifolin) | PTGS2 | 0.12730257 |
| 4',5-Dihydroxy-3,7-dimethoxy-6-methylflavone(8-Demethyllatifolin) | NAE1 | 0.12730257 |
| 4',5-Dihydroxy-3,7-dimethoxy-6-methylflavone(8-Demethyllatifolin) | ALOX5 | 0.12730257 |
| 4',5-Dihydroxy-3,7-dimethoxy-6-methylflavone(8-Demethyllatifolin) | NOX4 | 0.12730257 |
| 4',5-Dihydroxy-3,7-dimethoxy-6-methylflavone(8-Demethyllatifolin) | CA7 | 0.119403562 |
| 4',5-Dihydroxy-3,7-dimethoxy-6-methylflavone(8-Demethyllatifolin) | CA9 | 0.119403562 |
| 4',5-Dihydroxy-3,7-dimethoxy-6-methylflavone(8-Demethyllatifolin) | OPRM1 | 0.119403562 |
| 4',5-Dihydroxy-3,7-dimethoxy-6-methylflavone(8-Demethyllatifolin) | PTPN1 | 0.119403562 |
| 4',5-Dihydroxy-3,7-dimethoxy-6-methylflavone(8-Demethyllatifolin) | MMP9 | 0.119403562 |
| 4',5-Dihydroxy-3,7-dimethoxy-6-methylflavone(8-Demethyllatifolin) | PTPRS | 0.119403562 |
| 4',5-Dihydroxy-3,7-dimethoxy-6-methylflavone(8-Demethyllatifolin) | AMY1A | 0.119403562 |
| 4',5-Dihydroxy-3,7-dimethoxy-6-methylflavone(8-Demethyllatifolin) | ACHE | 0.119403562 |
| 4',5-Dihydroxy-3,7-dimethoxy-6-methylflavone(8-Demethyllatifolin) | CBR1 | 0.119403562 |
| 4',5-Dihydroxy-3,7-dimethoxy-6-methylflavone(8-Demethyllatifolin) | CDK5R1 | 0.119403562 |
| 4',5-Dihydroxy-3,7-dimethoxy-6-methylflavone(8-Demethyllatifolin) | MMP2 | 0.119403562 |
| 4',5-Dihydroxy-3,7-dimethoxy-6-methylflavone(8-Demethyllatifolin) | CA2 | 0.119403562 |
| 4',5-Dihydroxy-3,7-dimethoxy-6-methylflavone(8-Demethyllatifolin) | CA1 | 0.119403562 |
| 4',5-Dihydroxy-3,7-dimethoxy-6-methylflavone(8-Demethyllatifolin) | CDK1 | 0.119403562 |
| 4',5-Dihydroxy-3,7-dimethoxy-6-methylflavone(8-Demethyllatifolin) | AR | 0.119403562 |
| 4',5-Dihydroxy-3,7-dimethoxy-6-methylflavone(8-Demethyllatifolin) | GLO1 | 0.119403562 |
| 4',5-Dihydroxy-3,7-dimethoxy-6-methylflavone(8-Demethyllatifolin) | ARG1 | 0.119403562 |
| 4',5-Dihydroxy-3,7-dimethoxy-6-methylflavone(8-Demethyllatifolin) | TNKS2 | 0.111501865 |
| 4',5-Dihydroxy-3,7-dimethoxy-6-methylflavone(8-Demethyllatifolin) | TNKS | 0.111501865 |
| 4',5-Dihydroxy-3,7-dimethoxy-6-methylflavone(8-Demethyllatifolin) | ABCC1 | 0.111501865 |
| 4',5-Dihydroxy-3,7-dimethoxy-6-methylflavone(8-Demethyllatifolin) | KDM4E | 0.111501865 |
| 4',5-Dihydroxy-3,7-dimethoxy-6-methylflavone(8-Demethyllatifolin) | CA4 | 0.111501865 |
| 4',5-Dihydroxy-3,7-dimethoxy-6-methylflavone(8-Demethyllatifolin) | MMP12 | 0.111501865 |
| 4',5-Dihydroxy-3,7-dimethoxy-6-methylflavone(8-Demethyllatifolin) | APP | 0.111501865 |
| 4',5-Dihydroxy-3,7-dimethoxy-6-methylflavone(8-Demethyllatifolin) | PARP1 | 0.111501865 |
| 4',5-Dihydroxy-3,7-dimethoxy-6-methylflavone(8-Demethyllatifolin) | LCK | 0.111501865 |
| 4',5-Dihydroxy-3,7-dimethoxy-6-methylflavone(8-Demethyllatifolin) | MAOB | 0.111501865 |
| 4',5-Dihydroxy-3,7-dimethoxy-6-methylflavone(8-Demethyllatifolin) | TTR | 0.111501865 |
| 4',5-Dihydroxy-3,7-dimethoxy-6-methylflavone(8-Demethyllatifolin) | AHR | 0.111501865 |
| 4',5-Dihydroxy-3,7-dimethoxy-6-methylflavone(8-Demethyllatifolin) | CA12 | 0.111501865 |
| 4',5-Dihydroxy-3,7-dimethoxy-6-methylflavone(8-Demethyllatifolin) | AURKB | 0.111501865 |
| 4',5-Dihydroxy-3,7-dimethoxy-6-methylflavone(8-Demethyllatifolin) | CCNB3 | 0.111501865 |
| 4',5-Dihydroxy-3,7-dimethoxy-6-methylflavone(8-Demethyllatifolin) | BACE1 | 0.111501865 |
| 4',5-Dihydroxy-3,7-dimethoxy-6-methylflavone(8-Demethyllatifolin) | TERT | 0.111501865 |
| 4',5-Dihydroxy-3,7-dimethoxy-6-methylflavone(8-Demethyllatifolin) | DAPK1 | 0.111501865 |
| 4',5-Dihydroxy-3,7-dimethoxy-6-methylflavone(8-Demethyllatifolin) | EGFR | 0.111501865 |
| 4',5-Dihydroxy-3,7-dimethoxy-6-methylflavone(8-Demethyllatifolin) | KDR | 0.111501865 |
| 4',5-Dihydroxy-3,7-dimethoxy-6-methylflavone(8-Demethyllatifolin) | ALK | 0.111501865 |
| 4',5-Dihydroxy-3,7-dimethoxy-6-methylflavone(8-Demethyllatifolin) | ALOX15 | 0.111501865 |
| 4',5-Dihydroxy-3,7-dimethoxy-6-methylflavone(8-Demethyllatifolin) | MYLK | 0.111501865 |
| 4',5-Dihydroxy-3,7-dimethoxy-6-methylflavone(8-Demethyllatifolin) | SRC | 0.111501865 |
| 4',5-Dihydroxy-3,7-dimethoxy-6-methylflavone(8-Demethyllatifolin) | PIK3CG | 0.111501865 |
| 4',5-Dihydroxy-3,7-dimethoxy-6-methylflavone(8-Demethyllatifolin) | AXL | 0.111501865 |
| 4',5-Dihydroxy-3,7-dimethoxy-6-methylflavone(8-Demethyllatifolin) | XDH | 0.111501865 |
| 4',5-Dihydroxy-3,7-dimethoxy-6-methylflavone(8-Demethyllatifolin) | GPR35 | 0.111501865 |
| 4',5-Dihydroxy-3,7-dimethoxy-6-methylflavone(8-Demethyllatifolin) | MPG | 0.111501865 |
| 4',5-Dihydroxy-3,7-dimethoxy-6-methylflavone(8-Demethyllatifolin) | SLC22A12 | 0.111501865 |
| 4',5-Dihydroxy-3,7-dimethoxy-6-methylflavone(8-Demethyllatifolin) | CA6 | 0.111501865 |
| 4',5-Dihydroxy-3,7-dimethoxy-6-methylflavone(8-Demethyllatifolin) | CA14 | 0.111501865 |
| 4',5-Dihydroxy-3,7-dimethoxy-6-methylflavone(8-Demethyllatifolin) | CA13 | 0.111501865 |
| 4',5-Dihydroxy-3,7-dimethoxy-6-methylflavone(8-Demethyllatifolin) | CA5A | 0.111501865 |
| 4',5-Dihydroxy-3,7-dimethoxy-6-methylflavone(8-Demethyllatifolin) | NTRK2 | 0.111501865 |
| 4',5-Dihydroxy-3,7-dimethoxy-6-methylflavone(8-Demethyllatifolin) | MMP13 | 0.111501865 |
| 4',5-Dihydroxy-3,7-dimethoxy-6-methylflavone(8-Demethyllatifolin) | MMP3 | 0.111501865 |
| 4',5-Dihydroxy-3,7-dimethoxy-6-methylflavone(8-Demethyllatifolin) | TOP2A | 0.111501865 |
| 4',5-Dihydroxy-3,7-dimethoxy-6-methylflavone(8-Demethyllatifolin) | PIK3R1 | 0.111501865 |
| 4',5-Dihydroxy-3,7-dimethoxy-6-methylflavone(8-Demethyllatifolin) | CA3 | 0.111501865 |
| 4',5-Dihydroxy-3,7-dimethoxy-6-methylflavone(8-Demethyllatifolin) | MET | 0.111501865 |
| 4',5-Dihydroxy-3,7-dimethoxy-6-methylflavone(8-Demethyllatifolin) | CXCR1 | 0.111501865 |
| 4',5-Dihydroxy-3,7-dimethoxy-6-methylflavone(8-Demethyllatifolin) | CAMK2B | 0.111501865 |
| 4',5-Dihydroxy-3,7-dimethoxy-6-methylflavone(8-Demethyllatifolin) | NEK6 | 0.111501865 |
| 4',5-Dihydroxy-3,7-dimethoxy-6-methylflavone(8-Demethyllatifolin) | APEX1 | 0.111501865 |
| 4',5-Dihydroxy-3,7-dimethoxy-6-methylflavone(8-Demethyllatifolin) | NUAK1 | 0.111501865 |
| 4',5-Dihydroxy-3,7-dimethoxy-6-methylflavone(8-Demethyllatifolin) | AKR1C1 | 0.111501865 |
| 4',5-Dihydroxy-3,7-dimethoxy-6-methylflavone(8-Demethyllatifolin) | AKR1A1 | 0.111501865 |
| 4',5-Dihydroxy-3,7-dimethoxy-6-methylflavone(8-Demethyllatifolin) | SIGMAR1 | 0.111501865 |
| 4',5-Dihydroxy-3,7-dimethoxy-6-methylflavone(8-Demethyllatifolin) | CD38 | 0.111501865 |
| 4',5-Dihydroxy-3,7-dimethoxy-6-methylflavone(8-Demethyllatifolin) | GRK6 | 0.111501865 |
| 4',5-Dihydroxy-3,7-dimethoxy-6-methylflavone(8-Demethyllatifolin) | PLK1 | 0.111501865 |
| 4',5-Dihydroxy-3,7-dimethoxy-6-methylflavone(8-Demethyllatifolin) | BCHE | 0.111501865 |
| 4',5-Dihydroxy-3,7-dimethoxy-6-methylflavone(8-Demethyllatifolin) | ESRRA | 0.111501865 |
| 4',5-Dihydroxy-3,7-dimethoxy-6-methylflavone(8-Demethyllatifolin) | MCL1 | 0.111501865 |
| (7R,8S,8′S)-9-O-Benzoyl-isolariciresinol | ADRB3 | 0.120225751 |
| (7R,8S,8′S)-9-O-Benzoyl-isolariciresinol | PDK1 | 0.120225751 |
| (7R,8S,8′S)-9-O-Benzoyl-isolariciresinol | MMP9 | 0.120225751 |
| (7R,8S,8′S)-9-O-Benzoyl-isolariciresinol | SYK | 0.120225751 |
| (7R,8S,8′S)-9-O-Benzoyl-isolariciresinol | CDK2 | 0.120225751 |
| (7R,8S,8′S)-9-O-Benzoyl-isolariciresinol | CDK1 | 0.120225751 |
| (7R,8S,8′S)-9-O-Benzoyl-isolariciresinol | CDK4 | 0.120225751 |
| (7R,8S,8′S)-9-O-Benzoyl-isolariciresinol | ESR1 | 0.120225751 |
| (7R,8S,8′S)-9-O-Benzoyl-isolariciresinol | ADA | 0.120225751 |
| (7R,8S,8′S)-9-O-Benzoyl-isolariciresinol | MELK | 0.120225751 |
| (7R,8S,8′S)-9-O-Benzoyl-isolariciresinol | NR1H3 | 0.120225751 |
| (7R,8S,8′S)-9-O-Benzoyl-isolariciresinol | NR1H2 | 0.120225751 |
| (7R,8S,8′S)-9-O-Benzoyl-isolariciresinol | ITK | 0.120225751 |
| (7R,8S,8′S)-9-O-Benzoyl-isolariciresinol | BMP1 | 0.120225751 |
| (7R,8S,8′S)-9-O-Benzoyl-isolariciresinol | CCR1 | 0.120225751 |
| (7R,8S,8′S)-9-O-Benzoyl-isolariciresinol | RXRA | 0.120225751 |
| (7R,8S,8′S)-9-O-Benzoyl-isolariciresinol | HNF4A | 0.120225751 |
| (7R,8S,8′S)-9-O-Benzoyl-isolariciresinol | TOP1 | 0.120225751 |
| (7R,8S,8′S)-9-O-Benzoyl-isolariciresinol | ROCK2 | 0.120225751 |
| (7R,8S,8′S)-9-O-Benzoyl-isolariciresinol | ROCK1 | 0.120225751 |
| (7R,8S,8′S)-9-O-Benzoyl-isolariciresinol | NR3C1 | 0.120225751 |
| (7R,8S,8′S)-9-O-Benzoyl-isolariciresinol | P2RX3 | 0.120225751 |
| (7R,8S,8′S)-9-O-Benzoyl-isolariciresinol | CALCRL | 0.120225751 |
| (7R,8S,8′S)-9-O-Benzoyl-isolariciresinol | NOS2 | 0.120225751 |
| (7R,8S,8′S)-9-O-Benzoyl-isolariciresinol | ACVRL1 | 0.120225751 |
| (7R,8S,8′S)-9-O-Benzoyl-isolariciresinol | PDE2A | 0.120225751 |
| (7R,8S,8′S)-9-O-Benzoyl-isolariciresinol | HSP90AA1 | 0.120225751 |
| (7R,8S,8′S)-9-O-Benzoyl-isolariciresinol | MMP13 | 0.120225751 |
| (7R,8S,8′S)-9-O-Benzoyl-isolariciresinol | CXCR2 | 0.120225751 |
| (7R,8S,8′S)-9-O-Benzoyl-isolariciresinol | CXCR1 | 0.120225751 |
| (7R,8S,8′S)-9-O-Benzoyl-isolariciresinol | ROS1 | 0.120225751 |
| (7R,8S,8′S)-9-O-Benzoyl-isolariciresinol | BACE2 | 0.120225751 |
| (7R,8S,8′S)-9-O-Benzoyl-isolariciresinol | ADRA2C | 0.120225751 |
| (7R,8S,8′S)-9-O-Benzoyl-isolariciresinol | PDE10A | 0.120225751 |
| (7R,8S,8′S)-9-O-Benzoyl-isolariciresinol | PRKDC | 0.120225751 |
| (7R,8S,8′S)-9-O-Benzoyl-isolariciresinol | PIK3CA | 0.120225751 |
| (7R,8S,8′S)-9-O-Benzoyl-isolariciresinol | SLC6A4 | 0.120225751 |
| (7R,8S,8′S)-9-O-Benzoyl-isolariciresinol | RXRB | 0.120225751 |
| (7R,8S,8′S)-9-O-Benzoyl-isolariciresinol | RXRG | 0.120225751 |
| (7R,8S,8′S)-9-O-Benzoyl-isolariciresinol | AGTR1 | 0.120225751 |
| (7R,8S,8′S)-9-O-Benzoyl-isolariciresinol | LRRK2 | 0.120225751 |
| (7R,8S,8′S)-9-O-Benzoyl-isolariciresinol | PSEN2 | 0.120225751 |
| (7R,8S,8′S)-9-O-Benzoyl-isolariciresinol | ADAMTS5 | 0.120225751 |
| (7R,8S,8′S)-9-O-Benzoyl-isolariciresinol | ADAMTS4 | 0.120225751 |
| (7R,8S,8′S)-9-O-Benzoyl-isolariciresinol | TLR4 | 0.120225751 |
| (7R,8S,8′S)-9-O-Benzoyl-isolariciresinol | CTSD | 0.120225751 |
| (7R,8S,8′S)-9-O-Benzoyl-isolariciresinol | WEE1 | 0.120225751 |

**Table S3.** Molecular docking of the six components with different proteins.

| Components | Proteins | Uniprot | PDB | Docking binding energy | **Resolution** |
| --- | --- | --- | --- | --- | --- |
| Gaultherin | APP | P05067 | 1AAP | -7.1(kcal/mol) | 1.50 Å |
| Gaultherin | AMPK1 | Q13131 | 6C9H | -7.8(kcal/mol) | 2.65 Å |
| Gaultherin | AMPK/AMPK2 | P54646 | 2h6d | -7.4(kcal/mol) | 2.50 Å |
| Gaultherin | PIK3R1 | P27986 | 4JPS | -8.5(kcal/mol) | 2.2 Å |
| Gaultherin | PIK3CA | P42336 | 9ASF | -7.9(kcal/mol) | 1.77 Å |
| Gaultherin | PINK1 | Q9BXM7 | AF-Q9BXM7-F1 | -7.8(kcal/mol) | AlphaFold prediction |
| Dhasingreoside | APP | P05067 | 1AAP | -7.0(kcal/mol) | 1.50 Å |
| Dhasingreoside | AMPK1 | Q13131 | 6C9H | -7.3(kcal/mol) | 2.65 Å |
| Dhasingreoside | AMPK/AMPK2 | P54646 | 2h6d | -6.2(kcal/mol) | 2.50 Å |
| Dhasingreoside | PIK3R1 | P27986 | 4JPS | -9.8(kcal/mol) | 2.2 Å |
| Dhasingreoside | PIK3CA | P42336 | 9ASF | -8.7(kcal/mol) | 1.77 Å |
| Dhasingreoside | PINK1 | Q9BXM7 | AF-Q9BXM7-F1 | -9.1(kcal/mol) | AlphaFold prediction |
| GaultherosideA | APP | P05067 | 1AAP | -6.5(kcal/mol) | 1.50 Å |
| GaultherosideA | AMPK1 | Q13131 | 6C9H | -8.4(kcal/mol) | 2.65 Å |
| GaultherosideA | AMPK/AMPK2 | P54646 | 2h6d | -6.7(kcal/mol) | 2.50 Å |
| GaultherosideA | PIK3R1 | P27986 | 4JPS | -7.3(kcal/mol) | 2.2 Å |
| GaultherosideA | PIK3CA | P42336 | 9ASF | -7.5(kcal/mol) | 1.77 Å |
| GaultherosideA | PINK1 | Q9BXM7 | AF-Q9BXM7-F1 | -6.9(kcal/mol) | AlphaFold prediction |
| Gaultherin A | APP | P05067 | 1AAP | -6.3(kcal/mol) | 1.50 Å |
| Gaultherin A | AMPK1 | Q13131 | 6C9H | -7.3(kcal/mol) | 2.65 Å |
| Gaultherin A | AMPK/AMPK2 | P54646 | 2h6d | -6.2(kcal/mol) | 2.50 Å |
| Gaultherin A | PIK3R1 | P27986 | 4JPS | -8.1(kcal/mol) | 2.2 Å |
| Gaultherin A | PIK3CA | P42336 | 9ASF | -7.4(kcal/mol) | 1.77 Å |
| Gaultherin A | PINK1 | Q9BXM7 | AF-Q9BXM7-F1 | -7.5(kcal/mol) | AlphaFold prediction |
| Gaultherin B | APP | P05067 | 1AAP | -5.8(kcal/mol) | 1.50 Å |
| Gaultherin B | AMPK1 | Q13131 | 6C9H | -7.7(kcal/mol) | 2.65 Å |
| Gaultherin B | AMPK/AMPK2 | P54646 | 2h6d | -6.4(kcal/mol) | 2.50 Å |
| Gaultherin B | PIK3R1 | P27986 | 4JPS | -7.7 (kcal/mol) | 2.2 Å |
| Gaultherin B | PIK3CA | P42336 | 9ASF | -6.9 (kcal/mol) | 1.77 Å |
| Gaultherin B | PINK1 | Q9BXM7 | AF-Q9BXM7-F1 | -7.0 (kcal/mol) | AlphaFold prediction |
| (7R,8S,8′S)-9-O-Benzoyl-isolariciresinol | APP | P05067 | 1AAP | -6.4(kcal/mol) | 1.50 Å |
| (7R,8S,8′S)-10-O-Benzoyl-isolariciresinol | AMPK1 | Q13131 | 6C9H | -9.0(kcal/mol) | 2.65 Å |
| (7R,8S,8′S)-11-O-Benzoyl-isolariciresinol | AMPK/AMPK2 | P54646 | 2h6d | -6.4(kcal/mol) | 2.50 Å |
| (7R,8S,8′S)-12-O-Benzoyl-isolariciresinol | PIK3R1 | P27986 | 4JPS | -8.3(kcal/mol) | 2.2 Å |
| (7R,8S,8′S)-13-O-Benzoyl-isolariciresinol | PIK3CA | P42336 | 9ASF | -8.1(kcal/mol) | 1.77 Å |
| (7R,8S,8′S)-14-O-Benzoyl-isolariciresinol | PINK1 | Q9BXM7 | AF-Q9BXM7-F1 | -7.9 (kcal/mol) | AlphaFold prediction |
